# Molecular, Biological and Structural Features of V_L_ CDR-1 Rb44 Peptide, Which Targets the Microtubule Network in Melanoma Cells

**DOI:** 10.3389/fonc.2019.00025

**Published:** 2019-01-25

**Authors:** Natalia Girola, Pedro T. Resende-Lara, Carlos R. Figueiredo, Mariana H. Massaoka, Ricardo A. Azevedo, Rodrigo L. O. R. Cunha, Luciano Polonelli, Luiz R. Travassos

**Affiliations:** ^1^Department of Microbiology, Immunology and Parasitology, Experimental Oncology Unit, Federal University of São Paulo, São Paulo, Brazil; ^2^Computational Biology and Bioinformatics Laboratory, Federal University of ABC, Santo André, Brazil; ^3^Department of Molecular and Clinical Cancer Medicine, University of Liverpool, Liverpool, United Kingdom; ^4^Cancer Focus, São Paulo, Brazil; ^5^Chemical Biology Laboratory, Natural and Human Sciences Center, Federal University of ABC, Santo André, Brazil; ^6^Unit of Biomedical, Biotechnological and Translational Sciences, Department of Medicine and Surgery, Universitá degli Studi di Parma, Parma, Italy; ^7^Recepta Biopharma, São Paulo, Brazil

**Keywords:** metastatic melanoma, microtubule, tubulin, peptide, complementarity-determining region, apoptosis

## Abstract

Microtubules are important drug targets in tumor cells, owing to their role in supporting and determining the cell shape, organelle movement and cell division. The complementarity-determining regions (CDRs) of immunoglobulins have been reported to be a source of anti-tumor peptide sequences, independently of the original antibody specificity for a given antigen. We found that, the anti-Lewis B mAb light-chain CDR1 synthetic peptide Rb44, interacted with microtubules and induced depolymerization, with subsequent degradation of actin filaments, leading to depolarization of mitochondrial membrane-potential, increase of ROS, cell cycle arrest at G2/M, cleavage of caspase-9, caspase-3 and PARP, upregulation of Bax and downregulation of Bcl-2, altogether resulting in intrinsic apoptosis of melanoma cells. The *in vitro* inhibition of angiogenesis was also an Rb44 effect. Peritumoral injection of Rb44L1 delayed growth of subcutaneously grafted melanoma cells in a syngeneic mouse model. L1-CDRs from immunoglobulins and their interactions with tubulin-dimers were explored to interpret effects on microtubule stability. The opening motion of tubulin monomers allowed for efficient L1-CDR docking, impairment of dimer formation and microtubule dissociation. We conclude that Rb44 V_L_-CDR1 is a novel peptide that acts on melanoma microtubule network causing cell apoptosis *in vitro* and melanoma growth inhibition *in vivo*.

## Introduction

The polymerization dynamics of cytoskeleton molecules is crucial to the survival and to the energetic and mechanistic properties of cells and organisms. As important polymers in the mitotic process, microtubules are targets of anticancer drugs, with several compounds already being studied (1, 2).

Microtubule targeting agents (MTAs) exert inhibitory effects on cell proliferation, with cell cycle arrest at G2-M and induction of apoptosis (3). They may act as vascular-targeting drugs, disrupting microtubules in endothelial cells, which affects the blood supply in the tumor tissue (4). Microtubules also induce maturation and migration of dendritic cells, which are essential to the immune response (5).

MTAs can be divided into mechanistic acting categories as they either stabilize or destabilize microtubules (6). Microtubule-stabilizing agents such as paclitaxel and docetaxel bind to the taxane-binding site on β-tubulin, inhibiting microtubule depolymerization and intensifying its polymerization. Recently, Taxol/Paclitaxel has been described as first billion-dollar anticancer drug (7). Microtubule-destabilizing agents including colchicine and vinca alkaloid, typically bind to sites located at the intra-dimer interface and near the GTP binding site on β-tubulin, respectively. Such interactions induce inhibition of microtubule polymerization and promote depolymerization (8, 9). Although these agents are widely used in medicine, particularly paclitaxel and vinca alkaloids, drug resistance and side effects such as neurotoxicity, are significant limitations to MTAs clinical success (10, 11).

In the last decade, peptides displaying anticancer properties have been studied as promising alternative agents for cancer therapy (12, 13). Peptides are mostly non-genotoxic, have high affinity and selectivity for molecular targets on cancer cells, low cost production with feasible synthesis of derivatives, exhibiting low antigenicity and good tissue penetration (14, 15). Peptides can also be conjugated to large molecules to improve pharmacokinetics (16). Peptides can be displayed on the phage surface giving rise to specific sequences targeting different tissues or be developed from internal regions of transcription factors (17). Peptides and derivatives from natural sources such as marine animals and insects have been described with preferential antitumor activity without affecting normal cells (18, 19). Complementarity-determining regions (CDRs) of immunoglobulins (Igs) have been found to exhibit with high frequency, antiinfective, immunomodulatory, and antitumor activities (20–22).

Synthetic peptides corresponding to the Ig hypervariable CDRs, may display antitumor activities *in vivo*, as well as cytotoxic effects *in vitro* including cell cycle arrest, inhibition of tumor cell migration and invasion, induction of apoptosis, disruption of cytoskeleton dynamics (22–28), and many others.

We have previously described a novel bioactive mAb V_L_ CDR 1 peptide (C36L1), displaying *in vitro* and *in vivo* anti-tumor activities. Depolymerization of microtubules, leading to cytotoxic and cytostatic effects mediated by Rho-GTPase, PTEN, and PI3K/Akt signaling, have been characterized (26).

Presently, we investigated a V_L_ CDR1-derived synthetic peptide, Rb44, expressed in a anti-Lewis B monoclonal antibody, focusing on structural, biological and molecular docking properties, in comparison with two other V_L_ CDR1 peptides (Rb29L1 and C36L1), to understand the mechanism of action of Ig-CDR derived, apoptotic peptides targeting microtubules. Rb44L1 exerted both *in vitro* and *in vivo* anti-melanoma activities and inhibited endothelial cell sprouting *in vitro*.

## Materials and Methods

### Peptides

The L1 CDR amino acid sequences were obtained from the anti-Lewis B mAb antibody, V_L_ Rb44L1 (RSSQTITHGNGNTYLY-_NH2_), and from the anti-A34 mAb, V_L_ Rb29L1 (RSSTSLLHGNGNTYLT-_NH2_) according to Kabat et al. (29) CDR definition. The peptide sequences were purchased from Peptide 2.0 (Chantilly, VA) at 95–98% purity. All peptides were amidated at the C-terminus. Peptides were diluted in 1% DMSO-RPMI medium. In some experiments a scrambled Rb44L1 (Scr44) peptide was used (SIGTYSTRNYQHNLTG-_NH2_). The previously described C36L1 (KSSQSVFYSSNNKNYLA-_NH2_) was comparatively studied for molecular modeling.

### Tumor Cell Lines and Cell Culture

B16F10-Nex2 subline of murine melanoma cells was isolated at the Experimental Oncology Unit (UNONEX) of Federal University of São Paulo (UNIFESP) and registered in the Banco de Células do Rio de Janeiro (BCRJ), no. 0342. The original B16F10 cell line was obtained from the Ludwig Institute for Cancer Research (LICR), São Paulo Branch. Human melanoma cell line A2058; human carcinoma cell lines of colon, HCT-8; uterine cervix, SiHa; and breast, MCF-7; murine fibroblasts, 3T3-NIH; and human fibroblasts, GM637, were provided by the Ludwig Institute for Cancer Research and were a gift from Dr. Luiz F. Lima Reis (Hospital Sírio-Libanez, São Paulo). Human umbilical vein endothelial cells (HUVEC) were kindly provided by the Department of Immunology, Institute of Biomedical Sciences (University of São Paulo). Both cell lines were cultured at 37°C, under humid atmosphere and 5% CO_2_, in RPMI-1640 medium for tumorigenic cell lines and DMEM for non-tumorigenic ones, in both cases supplemented with 10 mM N-2-hydroxyethylpiperazine-N2 ethane sulfonic acid (HEPES), 24 mM sodium bicarbonate, 40 mg/L gentamicin, pH 7.2 and 10% fetal bovine serum (FBS).

### Cell Viability Assay

For IC_50_ determination, 1 × 10^4^ tumorigenic and non-tumorigenic cell lines were seeded in 96-well plates and treated at different concentrations ranging from 0 to 1 mM of Rb44L1 and Rb29L1 peptides for 24 h. Viable cells were quantified using the MTT (3-[4,5-dimethylthiazol-2-yl]-2,5-diphenyltetrazolium bromide) (Sigma-Aldrich, St. Louis, MO) assay. After incubation, 5 μL of MTT solution (5 mg/ml) was added to the cells, followed by incubation for 3 h at 37°C. Absorbance was measured in a microplate reader at 570 nm (SpectraMax-M2, Molecular Devices Software Pro 5.4, Sunnyvale, CA). IC_50_ was calculated using GraFit 5 data analysis software (Version 5.0.13).

### Chromatin Condensation and DNA Fragmentation Assays

Apoptotic melanoma cells treated with Rb44L1 peptide were examined by TUNEL staining, using the *in situ* Cell Death Detection Kit according with the manufacture's instruction (Roche Applied Science, Madison, WI). B16F10-Nex2 melanoma cells (1 × 10^4^) were seeded on 96-well clear-bottom black polystyrene microplate and incubated with 0, 130 and 260 μM of Rb44L1 peptide for 18 h. After incubation, cells were fixed in formaldehyde 2% for 20 min at room temperature, washed in PBS, and incubated with Hoechst 33342 (Invitrogen, Eugene, OR), at 10 μg/mL final concentration in the reaction buffer and TUNEL enzymatic substrate. Cells were washed and images were acquired and analyzed in a Cytell Cell image cytometer (GE Healthcare, Little Chalfont, UK).

### Annexin V and Propidium Iodide Labeling

B16F10-Nex2 cells (5 × 10^5^) were cultured in 6-well plates and further incubated with Rb44L1 at 0, 80 and 100 μM for 18 h at 37°C. After incubation, the Annexin V-FITC Apoptosis Detection Kit (Sigma-Aldrich, St. Louis, MO) was used and cells labeled with propidium iodide (PI) and FITC annexin V (AV) were analyzed by flow cytometry (BD Bioscience FACSCanto II equipment, Franklin Lakes, NJ), using FlowJo software (TreeStar Inc., Ashland, OR).

### Cell Cycle Analysis

B16F10-Nex2 (5 × 10^5^) cells were seeded in conical centrifugation tubes and incubated with 65 μM Rb44L1 peptide for 16 h in suspension. After incubation, the cells were washed with PBS and fixed in ethanol 70% for 1 h at 4°C. Cells were then washed again with PBS and stained with propidium iodide (PI) solution (50 μg/ml PI, 0.1 mg/ml RNAse A) for 20 min at 4°C in the dark. DNA fluorescence staining was acquired by FACSCalibur flow cytometer (Becton Dickinson, San Jose, CA). FlowJo software (Tree Star Inc., Ashland, OR) was used for post-acquisition analysis (20.000 events per sample). The microtubule depolymerizing CA4 (combretastatin A4, Sigma-Adrich, St. Louis, MO) was used at 75 μM as positive control of G2/M cell cycle arrest.

### Transmission Electron Microscopy

B16F10-Nex2 cells (1 × 10^6^) were seeded in 6-well plates. Cells were then incubated with peptide Rb44L1 at 260 μM for 18 h at 37°C. Fixation, dehydration and staining of the samples were performed as previously described (23). Jeol 1200 EXII electron microscope (Tokyo, Japan) was used for image acquisition.

### Mitochondrial Membrane Potential (Δψm)

B16F10-Nex2 cells (1 × 10^4^) were pre-incubated with the cationic lipophilic dye tetramethylrhodamine ethyl ester (TMRE) at 20 nM for 30 min, and then with peptide Rb44L1 at 0, 130, and 260 μM for 6 h. After the incubation period, images of living cells were acquired and analyzed by Cytell Cell Imaging System (GE Healthcare, Little Chalfont, UK).

### Superoxide Anion Measurement

Superoxide anion production was measured by dihydroethidium (DHE) assay. Briefly, 1 × 10^4^ cells cultivated on 96-well clear-bottom black plate were pre-incubated with DHE for 30 min at 37°C. Rb44L1 was added at 130 and 260 μM concentrations and fluorescence units were quantified after 16 h in a microplate reader (Molecular Devices M2, Sunnyvale, CA) adjusted for excitation at 370 nm and emission at 420 nm. As positive control, cells were treated with 5 mM of H_2_O_2_ at 37°C for 20 min, and the negative control run with no peptide.

### Cell Lysate Extracts and Western Blotting

B16F10-Nex2 cells (10^6^) were incubated with 0 and 130 μM of Rb44L1 peptide for different times (1, 3, 6, 8, and 24 h). After incubation, cells were washed in PBS and lysed with 300 μL of SDS sample buffer (62.5 mM Tris-HCl, pH 6.8 at 25°C, 2% w/v SDS, 10% glycerol, 50 mM DTT, 0.01% w/v bromophenol blue). Proteins from whole cell extracts were analyzed by Western blotting as previously described (20). The following primary, highly specific monoclonal antibodies, were used: rabbit anti-Bcl-2, -Bcl-xl, -Bax, -caspase-9 and cleaved caspase-9, -caspase-3 and cleaved caspase-3, -Parp and cleaved Parp, and -GAPDH (for total protein loading control), with secondary anti-rabbit IgG conjugated with horseradish peroxidase (HRP). All antibodies were purchased from Cell Signaling Technology (Beverly, MA) except for anti-GAPDH, acquired from Sigma-Aldrich (St. Louis, MO). Immunoreaction was revealed using the Luminata™ Forte solution (Millipore, Billerica, MA) and images were acquired using Uvitec Cambridge (Cambridge, UK). The molecular mass of each protein was estimated based on a pre-stained protein standard (Spectra Multicolor, ThermoScientific, Waltham, MA). Full-length Western blotting membranes are displayed in Figure S1.

### *In vitro* Angiogenesis Assay

The basement matrix Geltrex™ (Invitrogen, Eugene, OR) was added (30 μl/well) to coat a 96-well plate and allowed to polymerize for 40 min at 37°C. HUVEC cells (5 × 10^3^) suspended in RPMI medium supplemented with 0.2% of fetal calf serum were plated alone (control) or mixed with 5 μM of Rb44L1 peptide. The cells were incubated at 37°C for 6 h and images were captured with a microscope digital camera (Olympus, Tokyo, Japan). The numbers of pro-angiogenic structures (typically closed compartments or rings formed after endothelial cell sprouting) were counted from 3 different wells.

### Ethics Statement

The present study is part of Project 2010/51423-0 granted by the São Paulo State Research Support Foundation (FAPESP), Brazil. The protocols used for animal experiments were carried out in accordance with the Ethics Committee of Federal University of São Paulo, Brazil and have been approved via document CEP 1234/2011.

### Mice and Subcutaneous Melanoma Model

Eight-week-old male C57Bl/6 mice were acquired from the Center for Development of Experimental Models (CEDEME) at Federal University of São Paulo (UNIFESP), Brazil. The Ethics Committee for Animal Experimentation (UNIFESP) approved protocols of animal experiments. In the subcutaneous (s.c.) melanoma model, male C57Bl/6 mice (five per group) were subcutaneously grafted in the right flank with 1 × 10^5^ syngeneic B16F10-Nex2 melanoma cells. Animals were subjected to 5 peritumoral daily doses of 300 μg (total 10 mg/kg) of Rb44L1. DMSO (1%) in PBS, was the vehicle control. Treatment started after the tumor size reached 80 mm^3^ as measured with a caliper. The tumor volume (V) was calculated by the formula V = 0.52 × d^2^ × D, where d and D are short and long diameters of the tumor, respectively, measured every other day. Mice were euthanized at the end of experiments or when the tumor size reached the maximum allowed volume of 3,000 mm^3^.

### Live-Cell Imaging of Microtubule Dynamics

Real-time fluorescence microscopy of living B16F10-Nex2 melanoma cells previously modified by viral transduction for the expression of green fluorescent tubulin (CellLight® Reagents −2.0 BacMam, Life Technologies), was used to investigate the peptide interaction with microtubules. Viable green fluorescence protein (GFP) tubulin-expressing cells (1 × 10^4^) were incubated with Rb44L1 and Rb29L1 at 260 μM and fluorescent images were taken at 10-min intervals during 2 h using the time-lapse BioStation fluorescence microscope (Nikon Instruments, Inc, Melville, NY). For instance, humidity, temperature (37°C) and CO_2_ (5%) were carefully controlled. Fluorescence analysis and quantification were performed with the ImageJ software and the video was processed with the NIS-Elements analysis software (Nikon, Tokyo) and Adobe After Effects software.

### Fluorescence Staining of F-Actin

B16F10-Nex2 cells (5 × 10^4^) were seeded in 24-well microplates and incubated with different concentrations of Rb44L1 (0, 130 and 260 μM) for 30 min and 3 h. After incubation, cells were fixed in 3.7% of formaldehyde for 20 min at 4°C, blocked (1% BSA, 5% SFB, 0.1% Triton in 1X PBS) for 30 min at room temperature and stained with Hoechst 33342 (Invitrogen, Eugene, OR) and anti-phalloidin conjugated with FITC for 1 h at 37°C. Images were acquired and analyzed by Cytell Cell Imaging System (GE healthcare, Little Chalfont, UK).

### System Preparation and Molecular Dynamics

*De novo* peptide structure prediction was made by Pep-Fold3 webserver (30). We obtained the tubulin structure from PDB 4TV9 (31) (chains A and B). Protonation analysis was made by PROPKA3 (32). Energy minimization was carried out on GROMACS 5.1 (33) using CHARMM36 force field (34). Systems were built by CHARMM-GUI webserver (35, 36) with TIP3P water molecules (37) and counter ions, when charge balancing was required. Simulations consisted of 5,000 steps of steepest descent energy minimization, followed by 25 ps of NVT equilibration dynamics for L1-CDR peptides and 10 ns for tubulin. A NPT production molecular dynamics of 100 ns was carried out on GROMACS 5.1 using CHARMM36 force field for each system. Secondary structure assignment and hydrogen bonds (H-bonds) were analyzed by using VMD (38) plugins. H-bonds distance cut-off was set up at 3.0 Å with angle cut-off of 20°. All further MD analyses were made by GROMACS 5.1.

### Normal Mode Calculations and Generation of Low-Energy Conformations

Normal mode analysis (NMA) was performed using CHARMM c41b1 (39) and CHARMM36 force filed using DIMB (40) module and excluding CMAP (41). A distance dependent dielectric constant was employed to treat the electrostatic shielding by the solvent as described by Philot et al. (42). We used the mode 08 (open/close of tubulin monomers) as directional constraint to generate low-energy conformers along the mode trajectory using the VMOD algorithm in CHARMM as depicted by Louet et al. (43). The restraints were applied only on Cα atoms and the energy was computed for all atoms. The structures were displaced from 0.0 Å to +6.0 Å (open direction) using steps of 1.0 Å, resulting in 7 intermediate low-energy structures along the mode.

### Molecular Docking

In order to obtain different structures to perform molecular docking, we clustered the MD trajectory of each peptide. All MD frames were fitted to the reference structure and clustered with GROMOS method by using GROMACS 5.1, with a backbone RMSD cutoff of 2.0 Å for Rb29 and Rb44 and 5.0 Å for C36 (since the last is very flexible) resulting in 3, 11, and 8 different clusters, respectively. The center structure of each peptide cluster was then used in docking simulations, performed with Hex 8.0 (44). Hex depicts proteins as rigid bodies and makes a blind search through protein surface while it evaluates the interaction correlation by using the fast Fourier transformation algorithm. As described in Meissner et al. (45), solvation and desolvation effects were treated as surface phenomena, since the Hex algorithm models the interaction, excluding volume and complementarity of form. Approximately 350 solutions were found for each combination. We used BINANA 1.2 (46) as a rescore method to investigate the specific molecular basis guiding the interaction between tubulin and peptides.

### Chemiluminescent Dot-Blotting

Peptide Rb44L1 binding to microtubule structures was determined by chemiluminescent (CL) dot-blotting as described elsewhere (26) with some modifications. Peptides C36L1 (positive control), Rb44L1, scrambled-Rb44L1 (Scr44) at 10 μg/10 μL each, or vehicle (1% DMSO in milli-Q water), were applied on nitrocellulose membranes. They were blocked with 5% BSA in 0.05% PBS-Tween 20. B16F10-Nex2 cell protein lysate (50 μg/ml), prepared with non-denaturing protein extraction buffer according to the manufacturer's instructions (Cell Signaling, Beverly, MA), was applied onto the nitrocellulose membranes and incubated overnight at 4°C. After washing, membranes were incubated with anti-alpha tubulin antibody (Sigma-Aldrich, St. Louis, MO) for 1 h at 37°C followed by anti-rabbit IgG-HRP antibody for 1 h at 37°C. Immunoreactivity was determined using the Luminata™ Forte solution (Millipore, Billerica, MA). Images were acquired by Uvitec Cambridge (Cambridge, UK) with 1-min membrane exposure time. No reactivity with the control peptide was observed. To investigate the influence of GTP and Mn^2+^ on the peptide binding with α-tubulin, the membranes coated with 10 μg Rb44L1 or scrambled (Scr44) peptide were blotted with or without 1 mM GTP (Cytoskeleton, Denver, CO) and/or 1 mM Mn_2_SO${}_{4}^{.}$H_2_O (Sigma-Aldrich, St. Louis, MO) added to the cell lysate (50 μg/ml), for 2 h at 37°C. Chemiluminescence was detected as described above but with short membrane exposure time (20 s).

### Tubulin Polymerization Assay

Microtubule polymerization was evaluated using the Tubulin Polymerization Assay kit (Cytoskeleton, Inc., Denver, CO). Rb44L1 (130 μM) or Scr44 (130 μM); colchicine (50 μM); Rb44L1 (130 μM) + colchicine (50 μM), diluted with 1% DMSO in distilled water were added to 50 μl of the tubulin reaction mix with optimized volumes for inhibitor detection containing 2 mg/ml or 1 mg/ml of tubulin in 80 mM PIPES (piperazine-N-N'-bis [2- ethane sulfonic acid] sodium salt), pH 6.9, 2 mM MgCl_2_, 0.5 mM EGTA (ethylene glycol-bis N,N,N',N'- tetra acetic acid), 60% v/v glycerol, 1 mM GTP, and 10 μM of the fluorescent reporter. The black, flat bottom, half area 96-well plate, with the samples, was examined in a fluorescence microplate reader (SpectraMax-M2e, Molecular Devices, Sunnyvale, CA) every 1 min at 340 nm of excitation and 410 nm of emission for 40 or 180 min. To monitor the tubulin polymerization in the same condition as of the dot blotting assay, the reaction was prepared as described above with 2 mg/ml of purified tubulin in 0.1% of BSA in PBS and 3.4% of cell lysis buffer, without cell lysate.

### Statistical Analysis

The software GraphPad Prism 5.0 (San Diego, CA) was utilized for all tests. Statistical differences between groups were compared by Student's *t*-test. Differences in survival time and rate were evaluated by the Kaplan-Meier survival curves. *P-*values are indicated as ^*^p < 0.05, ^**^p < 0.01 and, ^***^p < 0.001.

## Results

### L1-CDR Peptides Differ in Dynamic Features

Peptides Rb44L1 and Rb29L1 were studied in comparison with peptide (C36L1), which exerts cytotoxicity by depolymerization of microtubules and displays antitumor activities, as previously investigated (26).

**Figure 1:**
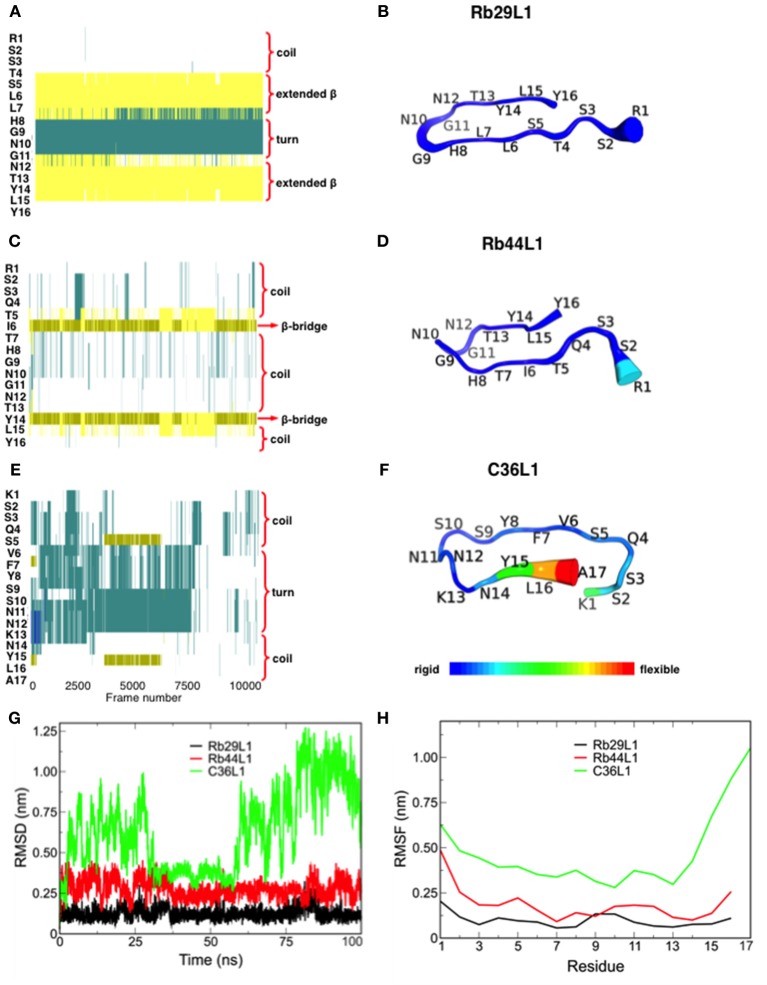
Secondary structure assignment during molecular dynamics and structural analysis of L1-CDRs. **(A,B)** Rb29L1 assumes a stable β-hairpin conformation during MD, showing a well established β-sheet between residues ^5^SLL and ^13^TYL; **(C,D)** Rb44L1 shows a recurrent β-bridge between residues ^5^TI and ^14^YL; **(E,F)** C36L1 presents the most flexible conformation, in its majority composed by turn and coil. Secondary structure color code: turn, in green; extended conformation (β-sheet), in yellow; isolated bridge, in gold; 3-10 helix, in blue; coil, in white; **(G)** root-mean-squared deviation of backbone atoms of Rb29L1, Rb44L1, and C36L1. Rb29L1 remains nearly at the same conformation during all MD, an effect also seen for Rb44L1, although with less intensity. C36L1, nonetheless, presented a great conformational variation; **(H)** root-mean-squared fluctuations of Cα atoms of Rb29L1, Rb44L1 and C36L1. Cα fluctuation, or flexibility, follows RMSD pattern. Rb29L1 presents a rigid structure while Rb44L1 and C36L1 are more flexible, the latter more pronounced.

In spite of the sequence similarity, the dynamics of L1-CDRs were very different from each other. Rb29L1 assumed a stable β-hairpin conformation, with residues ^5^SLL and ^13^TYL forming the β-sheet (Figures 1A,B). In turn, Rb44L1 showed only an intermittent β-bridge between residues ^5^TI and ^14^YL (Figures 1C,D). C36L1, however, did not assume any ordered structure (Figures 1E,F). Root-mean-squared deviation (RMSD) of backbone heavy atoms and Cα root-mean-squared fluctuation (RMSF) calculations were performed to evaluate structure stability along the molecular dynamics (MD). Results confirmed the stability of Rb29L1, while C36L1 showed several conformational shifts (Figure 1G). Flexibility analysis confirmed this profile (Figure 1H). H-bonds formation during the dynamics could address these structural differences among the peptides. Rb29L1 showed more internal H-bonds than the other peptides, therefore it is more rigid. Table 1 summarizes these interactions. The trajectories of each peptide MD were clustered, according to RMSD, onto representative conformations to perform docking simulations (Figure S2).

**Table 1:** Hydrogen bonds formation during molecular dynamics of L1-CDR peptides^*^.

**Hydrogen bonds**	**Occupancy (%)**
***Rb29L1***	
ARG1-Side-NH1 – TYR16-Side-OT1	34.94
ARG1-Side-NH1 – TYR16-Side-OT2	17.56
ARG1-Side-NH2 – TYR16-Side-OT1	17.78
ARG1-Side-NH2 – TYR16-Side-OT2	37.64
SER3-Side-OG – TYR16-Side-OT1	27.63
SER3-Side-OG – TYR16-Side-OT2	18.97
THR4-Main-N – TYR16-Side-OT1	11.7
THR4-Main-N – TYR16-Side-OT2	27.13
LEU6-Main-N – TYR14-Main-O	42.05
HIS8-Main-N – ASN12-Main-O	43.21
TYR14-Main-N – LEU6-Main-O	36.03
TYR16-Main-N – THR4-Main-O	43.64
TYR16-Side-OH – HIS8-Side-NE2	17.83
***Rb44L1***	
THR7-Main-N – THR13-Main-O	36.35
LEU15-Main-N – THR5-Main-O	54.47
***C36L1***	
ALA17-Main-N – GLN4-Main-O	11.51

* *Only interactions with ≥ 10% occupancy are shown*.

### *In vitro* Cytotoxicity of CDR Peptides

We investigated the anti-tumor potential of two L1-CDR-derived peptides: Rb44L1 from anti-Lewis B mAb and Rb29L1 from anti-A34 mAb. The IC_50_ values were determined for the Rb44L1 and Rb29L1 against different tumorigenic and non-tumorigenic cell lines (Table 2). Peptide Rb44L1 showed the lowest IC_50_ values as compared to Rb29L1. The concentrations of 130 μM (IC_50_) and 260 μM (IC_100_), respectively, were therefore used in the subsequent experiments with B16F10-Nex2 melanoma cells. Rb44L1, was less active against non-tumorigenic cells, including murine and human fibroblasts, 3T3-NIH and GM637 cell lines. In the concentration range of 0 to 0.140 mM, no cytotoxicity was observed in these cells. Rb29L1 IC_50_ values were 3- to 10-fold higher than those of Rb44L1 in tumorigenic cell lines.

**Table 2:** IC_50_ values of the bioactive peptide Rb44L1 and control Rb29L1 against tumorigenic and non-tumorigenic lineages after 16 h of incubation.

**Cell lineages**	**IC**_****50****_ **(μM) ± SD**	
	**Rb44L1**	**Rb29L1**
B16F10-Nex2	130 ± 5.8	465 ± 67
A2058	66 ± 2.0	265 ± 16
MCF-7	134 ± 2.4	858 ± 53
SIHA	51 ± 6.6	773 ± 61
HCT-8	81 ± 1.5	821 ± 57
3T3-NIH^*^	>140	>140
GM637^*^	>140	>140

* *Non-tumorigenic cell lines*.

### Rb44L1 Induces Apoptosis

**Figure 2:**
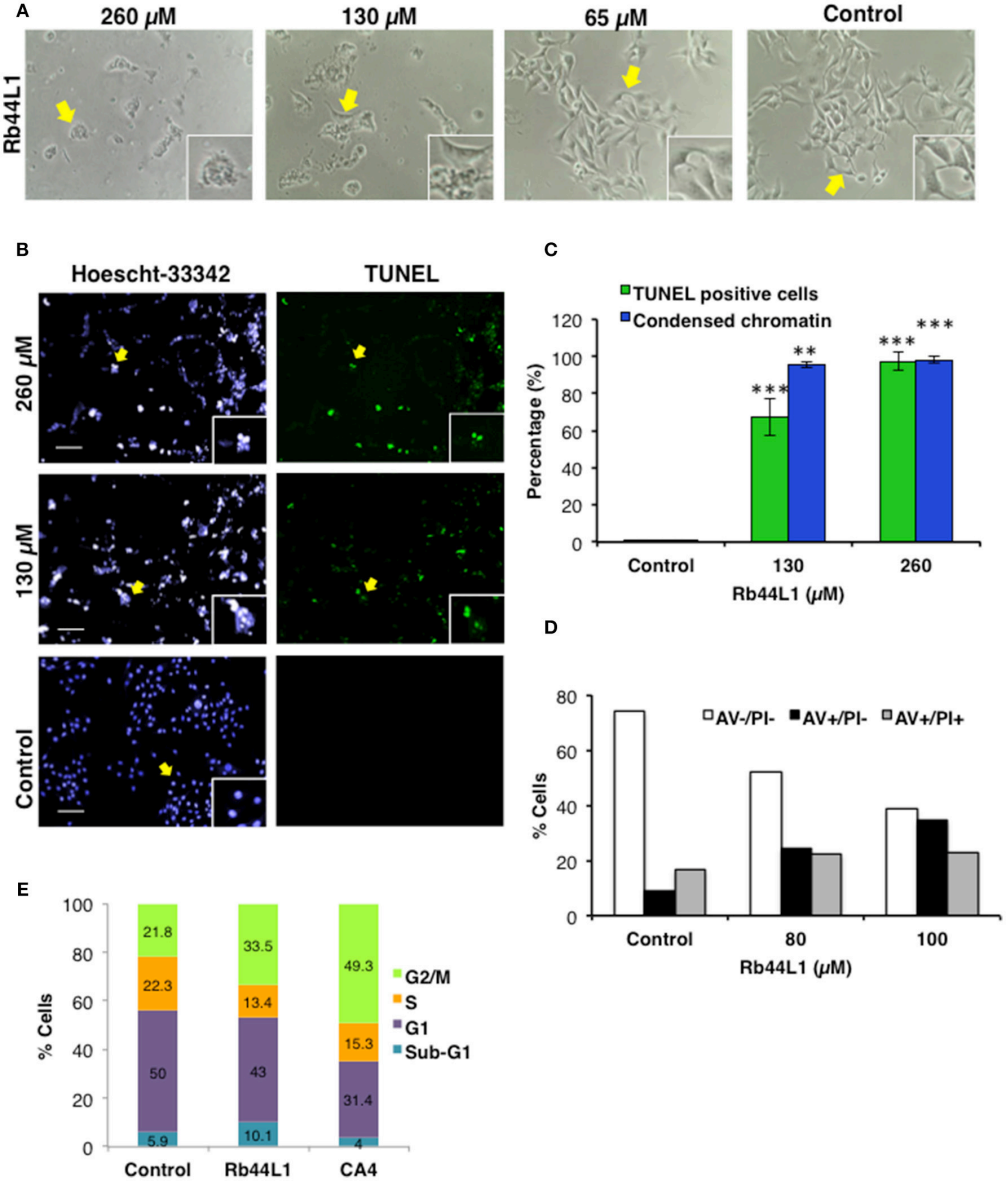
Rb44L1 induces apoptosis in melanoma cells. **(A)** morphological changes were analyzed by light microscopy. Representative images of cells treated with different doses of Rb44L1 or untreated cells (control). Arrows indicate inserts (x200, magnification); **(B)** representative images of chromatin condensation (Hoescht 33342, blue) and DNA fragmentation (TUNEL, green) of tumor cells treated with different concentrations of Rb44L1 for 18 h. Scale bar represents 50 μm; **(C)** percentage of TUNEL positive cells and condensed nuclei. ^**^p < 0.01 and ^***^p < 0.001 in comparison to untreated cells; **(D)** percentage of apoptotic cells determined by the externalization of phosphatidylserine; **(E)** cell cycle of B16F10-Nex2 cells after incubation with Rb44L1 at 65 μM for 16 h. Percent tumor cells at Sub-G1, G1, S, and G2/M phases are indicated. CA4 was used as positive control.

Changes in the dynamics of the cytoskeleton have been implicated in the induction of apoptosis. Here, we show that Rb44L1 induced morphological alterations typical of apoptotic cell death such as cellular shrinkage, membrane blebs and cell rounding-up with pseudopodia retraction in B16F10-Nex2 melanoma cells when incubated with peptide at IC_50_ (130 μM) and IC_100_ (260 μM) for 18 h (Figure 2A). Chromatin condensation was observed in 95% and 98% of tumor cells treated with Rb44L1 at 130 and 260 μM, respectively, for 18 h. DNA fragmentation was determined by green positive TUNEL staining in B16F10-Nex2 cells treated with 130 and 260 μM of Rb44L1 (Figures 2B,C). Both DNA condensation and fragmentation were significantly higher in Rb44L1-treated cells as compared with the negative control (for chromatin condensation, ^**^p < 0.01 at 130 μM, ^***^p < 0.001 at 260 μM; and for DNA fragmentation, ^***^p < 0.001 at both concentrations). Additionally, we observed that Rb44L1 could significantly enhance the translocation of phosphatidylserine (PS) to the outer leaflet of the plasma membrane, indicating early apoptosis. We observed a significant increase in the number of early apoptotic events in cells treated with Rb44L1 at 80 and 100 μM, in comparison with untreated control cells (Figure 2D). Finally, Rb44L1 inhibited cell proliferation with cell cycle arrest, at 65 μM (Figure 2E). The S-phase area decreased from 22.3 to 13.4%, with increase of the G2/M phase (from 21.8 to 33.5%). Microtubule depolymerizing combretastatin-A4 was used as positive control.

### Morphological and Functional Alterations in Mitochondria and ROS Production

**Figure 3:**
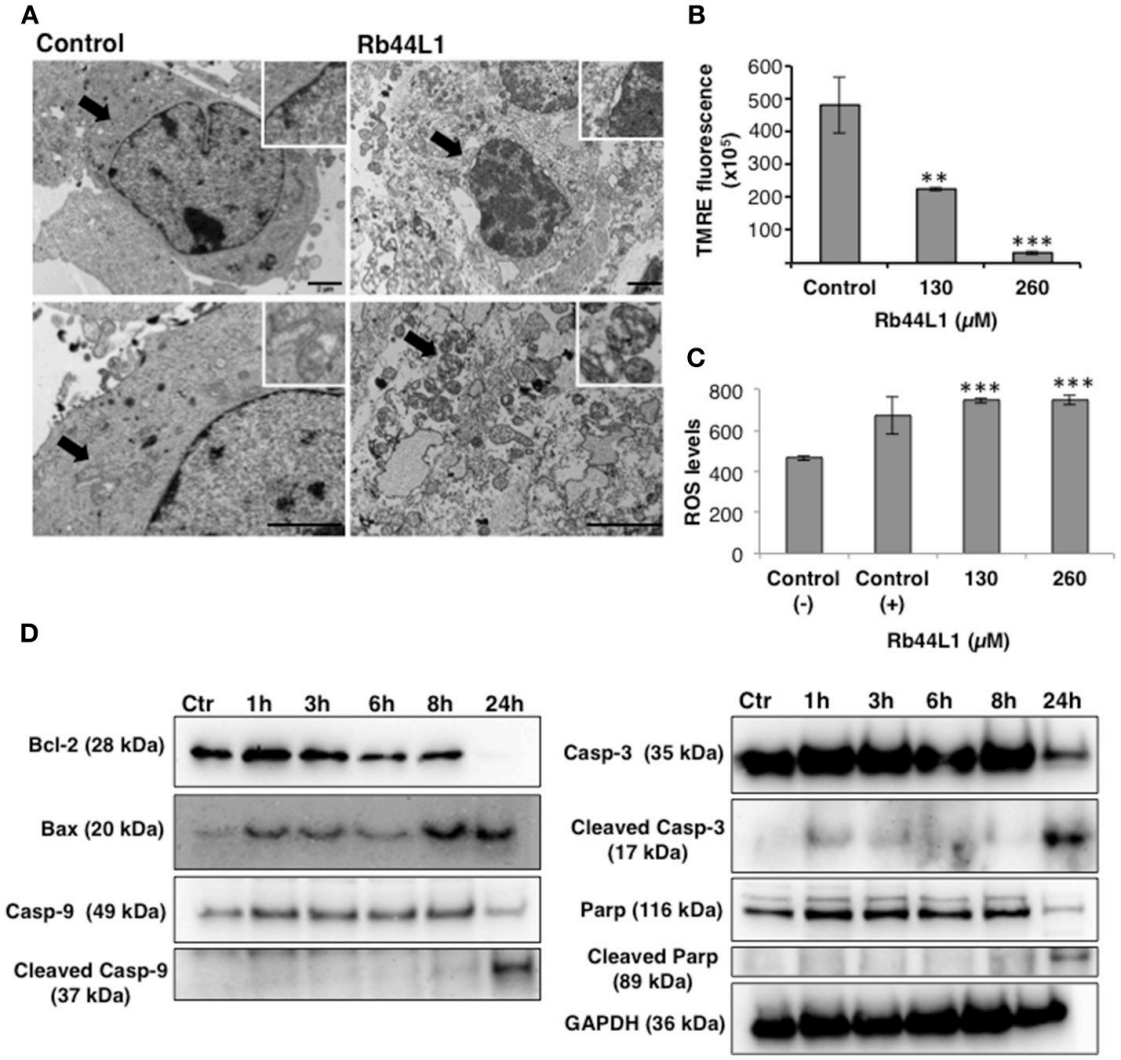
Rb44L1 induces morphological alterations in mitochondria. **(A)** B16F10-Nex2 cells were treated with 260 μM of Rb44L1 for 18 h and examined by transmission electron microscopy. Representative micrographs of untreated cells (control) and Rb44L1 treated cells. Arrows indicate mitochondrial ultrastructure in the inserts; scale bar represents 2 μm; **(B)** loss of mitochondrial transmembrane potential in B16F10-Nex2 cells treated with 130 and 260 μM of Rb44L1 for 6 h, probed with red TMRE. **p < 0.01 and ***p < 0.001 in comparison to the control; **(C)** enhanced superoxide anion production observed by DHE staining in B16F10-Nex2 cells treated with different concentrations of Rb44L1 for 16 h, vehicle control (Control −) and 5 mM H_2_O_2_ as positive control (Control +). The conversion of DHE to ethidium by oxidation was acquired at 370 nm (excitation) and 420 nm (emission). ***p < 0.001 in relation to control (−); **(D)** levels of apoptosis related proteins in Rb44L1-treated melanoma cells. Time-dependent effect on cell signaling of B16F10-Nex2 incubated with Rb44L1 at 130 μM. Levels of total and cleaved caspase-3,−9, cleaved PARP, Bax, Bcl-2, and Bcl-xl during Rb44-induced apoptosis are shown by Western blotting. GAPDH was used as loading control. A single cell-lysate sample was used in the same experiment and the Western blotting membranes were processed in parallel for antibody reactivity. Uncropped, full-length blottings are shown in Figure S1.

Transmission electron microscopy (TEM) of Rb44L1-treated B16F10-Nex2 cells, at 260 μM for 18 h, showed condensed chromatin, nuclear membrane detachment, enlarged, and vacuolated mitochondria with damaged cristae surrounded by heavily injured cytoplasmic organelles compared to untreated cells (Figure 3A). The collapse of the mitochondria transmembrane potential (Δψm) was observed on early incubation with Rb44L1 (0, 130, and 260 μM). After 6 h, reduction of TMRE fluorescence (53 and 94% reduction in cells treated with 130 and 260 μM, respectively; ^**^p < 0.01 and ^***^p < 0.001 in relation to untreated cells) was observed indicating mitochondrial damage in these cells (Figure 3B). Tumor cells were incubated with Rb44L1 at 130 and 260 μM for 16 h and ROS levels were detected using DHE dye measured by fluorimetry. Hydrogen peroxide (H_2_O_2_) was used as positive control (Control +) at 5 mM. Accumulation of ROS (59% in relation to untreated cells; ^***^p < 0.001) was observed in cells treated with Rb44L1 at both concentrations (Figure 3C).

### Rb44L1 Elicited Caspase Activation

Different pro- and anti-apoptotic proteins in total cell lysates were evaluated by Western blotting in Rb44L1-treated B16F10-Nex2 cells at 130 μM and different incubation periods. We observed that Rb44L1 induced early increase of pro-apoptotic Bax protein, followed by the cleavage of caspase-9, caspase-3 and PARP, together with downregulation of anti-apoptotic protein Bcl-2 (Figure 3D). GAPDH was used as loading control.

### Rb44L1 Inhibited Angiogenesis *in vitro*

**Figure 4:**
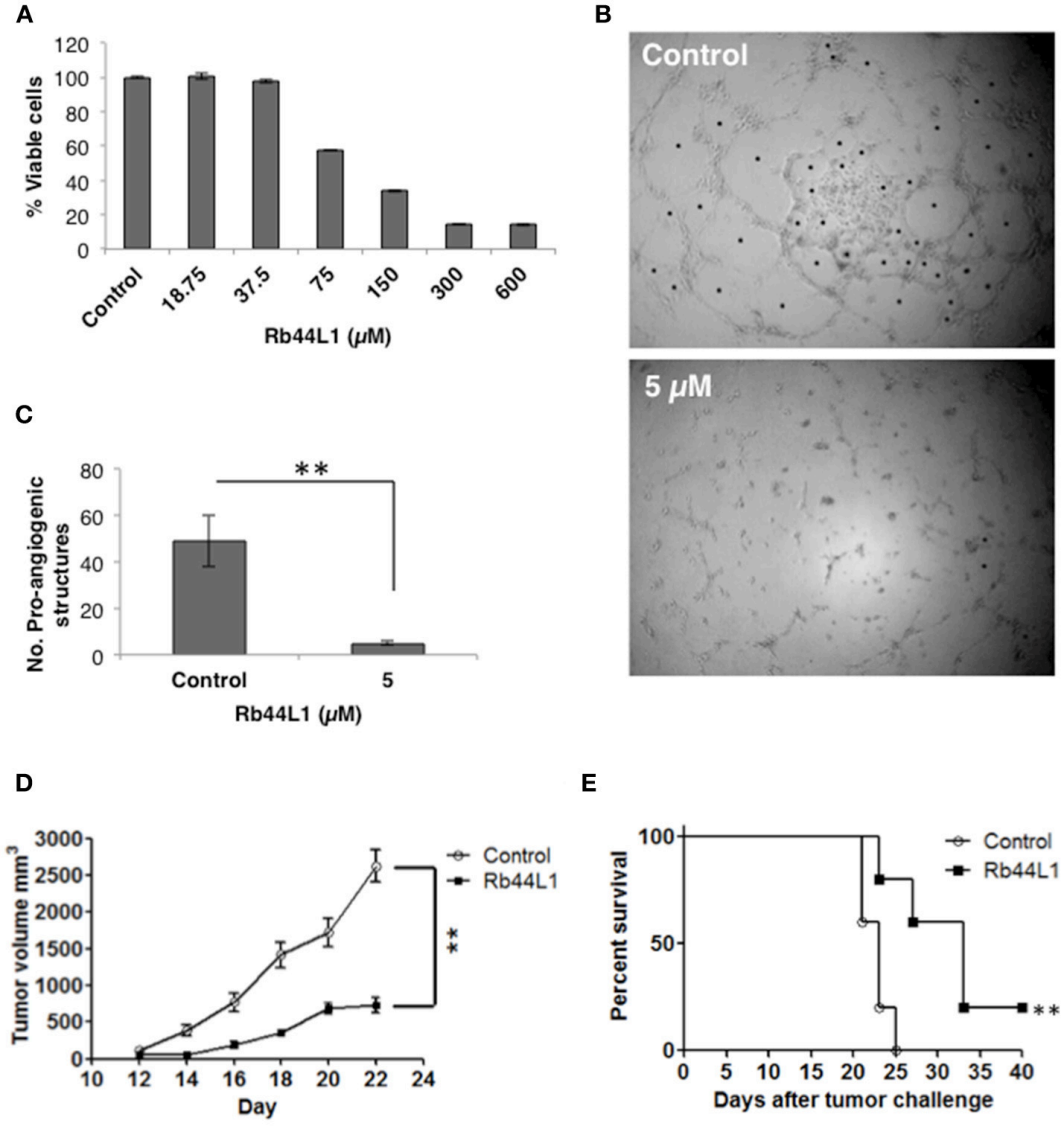
Rb44L1 inhibits HUVEC sprouting on Geltrex™ Matrix. **(A)** dose-response curve of Rb44L1 on HUVEC cells; **(B,C)** Inhibition by Rb44L1 (5 μM) on HUVEC sprouting on Geltrex™ Matrix to form closed proangiogenic structures; **p < 0.01 compared to untreated control. Rb44L1 prevents tumor progression. **(D)** 1 × 10^5^ syngeneic B16F10-Nex2 cells were subcutaneously injected in C57Bl/6 mice. Peritumoral daily doses of 300 μg of Rb44L1 peptide were administered during five consecutive days. Tumor volume was measured and documented during the treatment period. **p < 0.01 in comparison with control group treated with PBS; **(E)** survival of C57Bl/6 challenged mice after treatment with Rb44L1 or PBS (control). **p < 0.01 in relation to control group.

The cytotoxicity of Rb44L1 at different concentrations was assayed in the HUVEC lineage (Figure 4A). A non-cytotoxic concentration was used for the inhibition of endothelial cell (HUVEC) sprouting in Geltrex™ Matrix. Rb44L1 at 5 μM for 6 h, significantly inhibited 90% of endothelial cell sprouting, with the number of compartments built by intercellular connections being compared to that of the control (^**^p < 0.01; Figures 4B,C).

### Antitumor Activity *in vivo* Against Subcutaneous Melanoma

The *in vivo* antitumor activity was also investigated in a subcutaneously grafted, syngeneic murine melanoma model. Peritumoral injections of Rb44L1 at 15 mg/Kg significantly delayed tumor volume progression (^**^p < 0.01), and also prolonged mice survival (^**^p < 0.01) (Figures 4D,E). Mice were euthanized at the scheduled end of experiments, or before, should the tumors ulcerate or reach the maximum allowed volume of 3,000 mm^3^.

### Rb44L1 Interacts With Microtubules and Induces Cytoskeleton Disruption in Melanoma Cells

**Figure 5:**
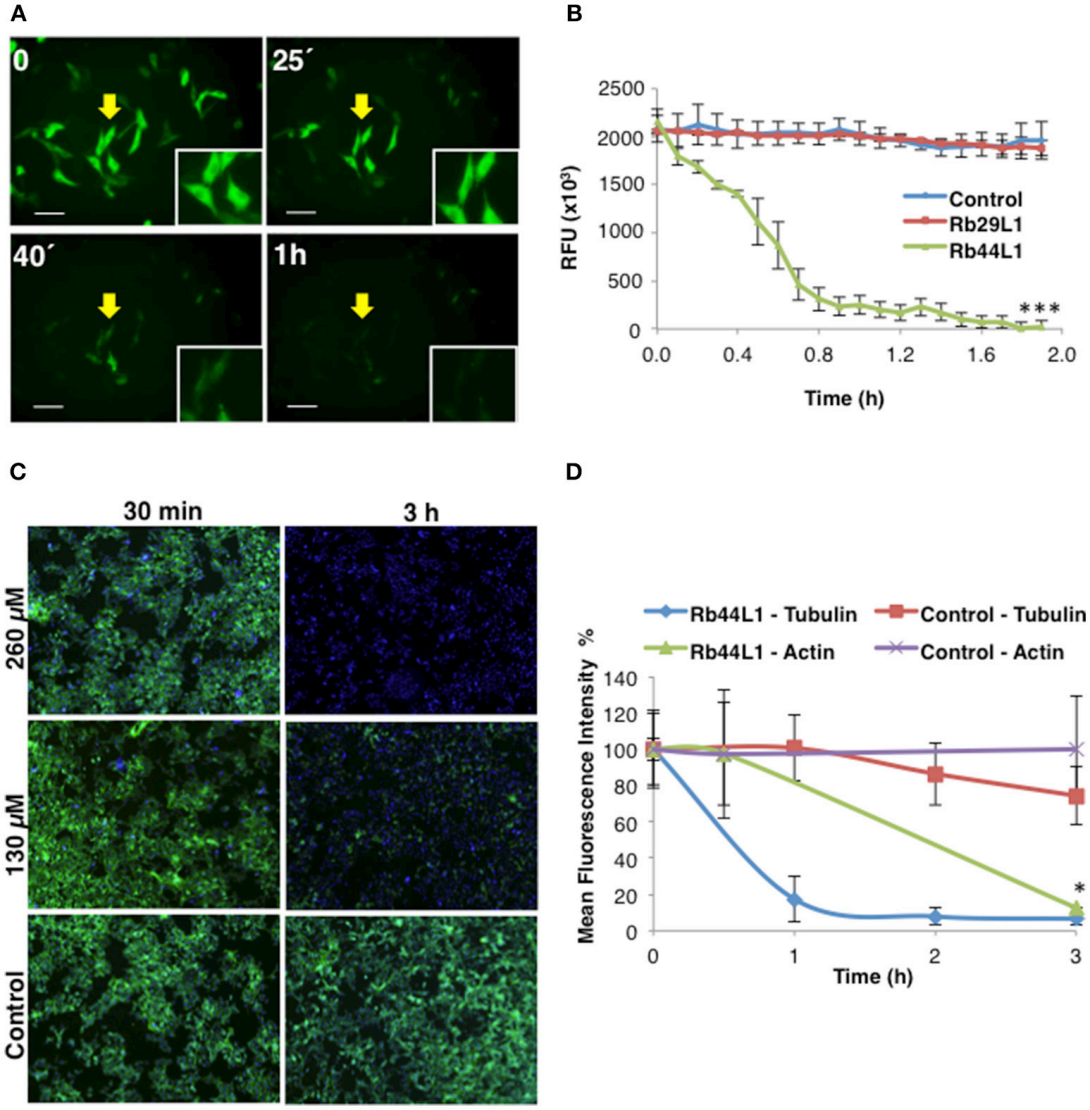
Rb44L1 targets microtubules and disrupts tubulin assembly. **(A)** B16F10-Nex2 cells expressing baculovirus-transduced fluorescent tubulin were incubated with Rb44L1 at 260 μM; representative image of microtubule integrity is shown. Scale bar represents 50 μm; **(B)** microtubule dissociation was quantified in Rb44L1 and Rb29L1 treated cells and expressed as fluorescence decreased intensity and complete dispersion. ***p < 0.001 in comparison to untreated cells; **(C)** representative images of B16F10-Nex2 cells treated for different times with 130 and 260 μM of Rb44L1. Merged images of phalloidin-FITC and Hoescht 33342 staining are shown; **(D)** loss of actin and tubulin assembly integrity in Rb44L1 treated cells was quantified and compared. Results are expressed by fluorescence intensity. *p < 0.05 comparing microtubules and actin disruption.

Disruption of the microtubule integrity in B16F10-Nex2 cells was monitored during the incubation with Rb44L1 and Rb29L1. Microtubules were assessed by live-cell imaging using B16F10-Nex2 cells previously transduced with a genetic modified insect virus (baculovirus) containing a tubulin-green fluorescent fusion-protein construct (CellLight®, Life Technologies). The fluorescence of live murine melanoma cells was monitored and quantified for 2 h during incubation with 260 μM of Rb44L1 and Rb29L1. The Rb44L1 peptide drastically reduced microtubule fluorescence compared to the negative control (Figures 5A,B), indicating that the microtubule network was depolymerized during the incubation with Rb44L1, whereas no depolymerization was seen in Rb29L1 treated cells. A representative video showing the kinetics of microtubule depolymerization in B16F10-Nex2 cells during the incubation with Rb44L1 and Rb29L1 is available in Video S1.

In addition to investigating whether Rb44L1 would also affect the integrity of F-actin, the reaction was assessed simultaneously using a phalloidin-FITC probe, as described in methods. We observed that F-actin integrity was completely lost after 3 h of incubation with Rb44L1 at 260 and 130 μM (Figure 5C). Actin degradation occurred after the microtubule disruption process, as evidenced in the cytoskeleton integrity quantification analysis (Figure 5D), suggesting that actin filaments were disrupted as a consequence of microtubule depolymerization (^*^p < 0.05 comparing microtubule and actin disruption). Less than 55 or 65% of cytotoxicity was seen when testing both concentrations of Rb44L1 at 260 and 130 μM, respectively, in the first hours of incubation (Figure S3).

### Normal Modes Expose Nonexchangeable Nucleotide and Colchicine Binding Sites

**Figure 6:**
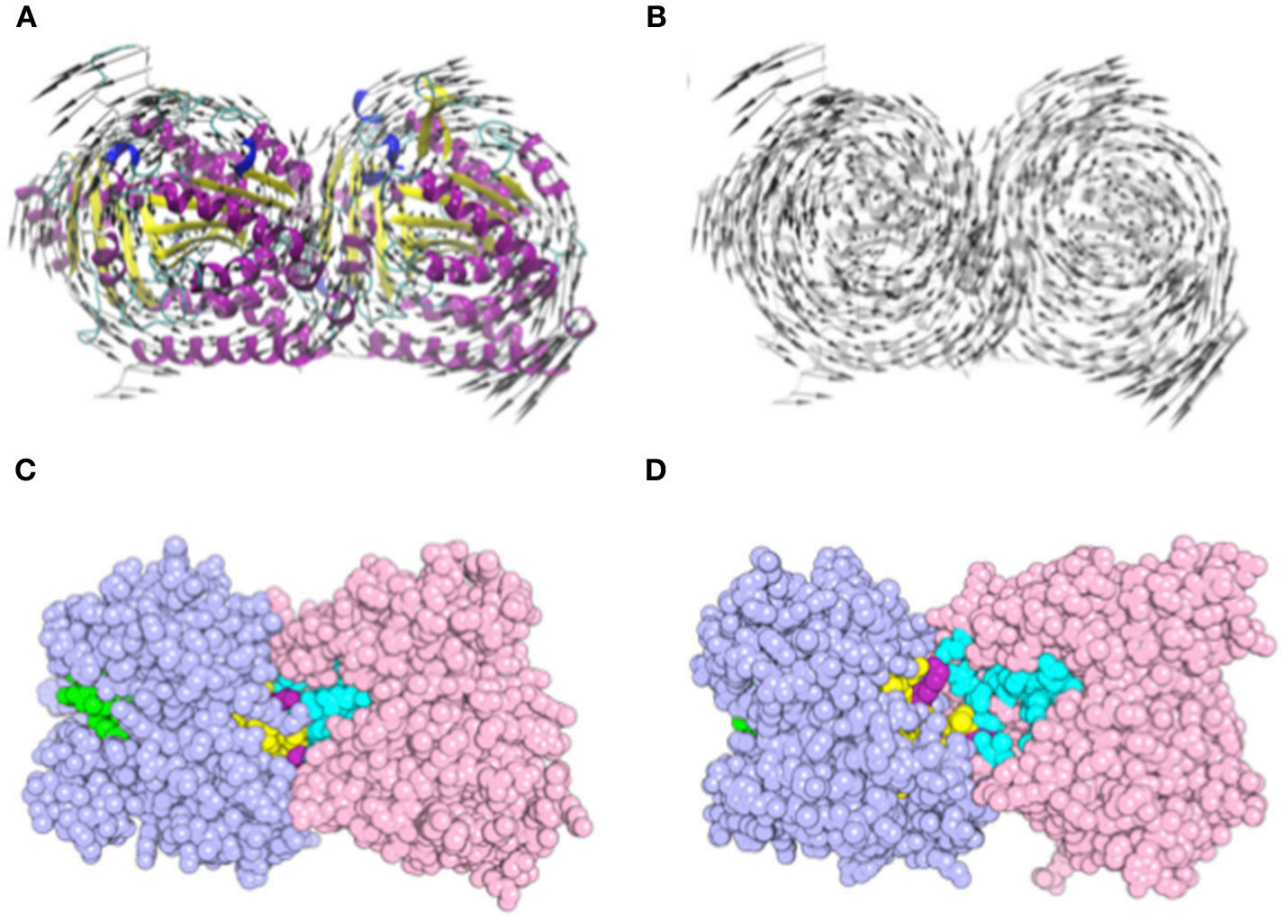
Motion representation of normal mode 8 and nucleotide/colchicine site exposition as a result of α/β-tubulin displacement. **(A)** cartoon representation of α/β-tubulin normal mode 8. The circular motion in opposite directions of each tubulin monomer promotes the exposition of a nucleotide and colchicine binding sites; **(B)** highlight of vector directions. Vectors are placed into Cα atoms of each residue. Secondary structure color code: turn in green; β-sheet in yellow; β-bridge in gold; α-helix in purple; G, 3-10 helix in blue; and C, Coil in white. **(C,D)** α/β-tubulin crystallographic structure (PDB 4TV9) where atoms are represented as spheres. **(C)** comparison of GTP N-site (cyan), colchicine (yellow), and GTP E-site (green) site exposition between **(C)** α/β-tubulin crystallographic structure (PDB 4TV9); and **(D)** α/β-tubulin displaced by 6 Å along normal mode 8. Atoms are represented as spheres and residues present in both colchicine, and GTP N-site are colored in purple. α-Tubulin is represented in light-pink and β-tubulin in light-blue.

Normal mode analysis (NMA) was employed to investigate the opening motion of tubulin monomers. We hypothesized that this opening motion would be required to expose the nucleotide binding site located at α-tubulin (N-site) and dimer interface. Such exposition could favor the efficient docking of L1-CDR peptides and impair the tubulin dimer assembly, finally leading to microtubule dissociation. This motion was verified as the normal mode 8 (Figures 6A,B). Using the VMOD routine implemented on CHARMM, we performed a mass-weighted displacement of tubulin structure along mode 8, to produce energy-relaxed structures with gradually exposed nucleotide site. Tubulin residues originally in contact with GTP (contacts within 4.5 Å) showed a solvent-accessible surface area (SASA) of 511 Å2 at the crystallographic structure (PDB 4TV9), while the same residues were more exposed after a displacement of 6 Å, presenting a SASA of 588 Å2 (Figures 6C,D). The same occurred for the colchicine site, which presented a SASA of 225 Å2 before the displacement and 236 Å2 after mass-weighted displacement of 6 Å.

### Docking Studies Reveal the Importance of Electrostatic Interactions

Docking calculations were performed using 7 tubulin structures generated from NMA displacement against the central structure of each L1-CDR peptide cluster (3 for Rb29L1, 11 for Rb44L1, and 8 for C36L1). In every docking round, an average of 350 different solutions was calculated. We then evaluated the best solution from Hex with BINANA to better understand the key binding characteristics governing the interaction.

**Figure 7:**
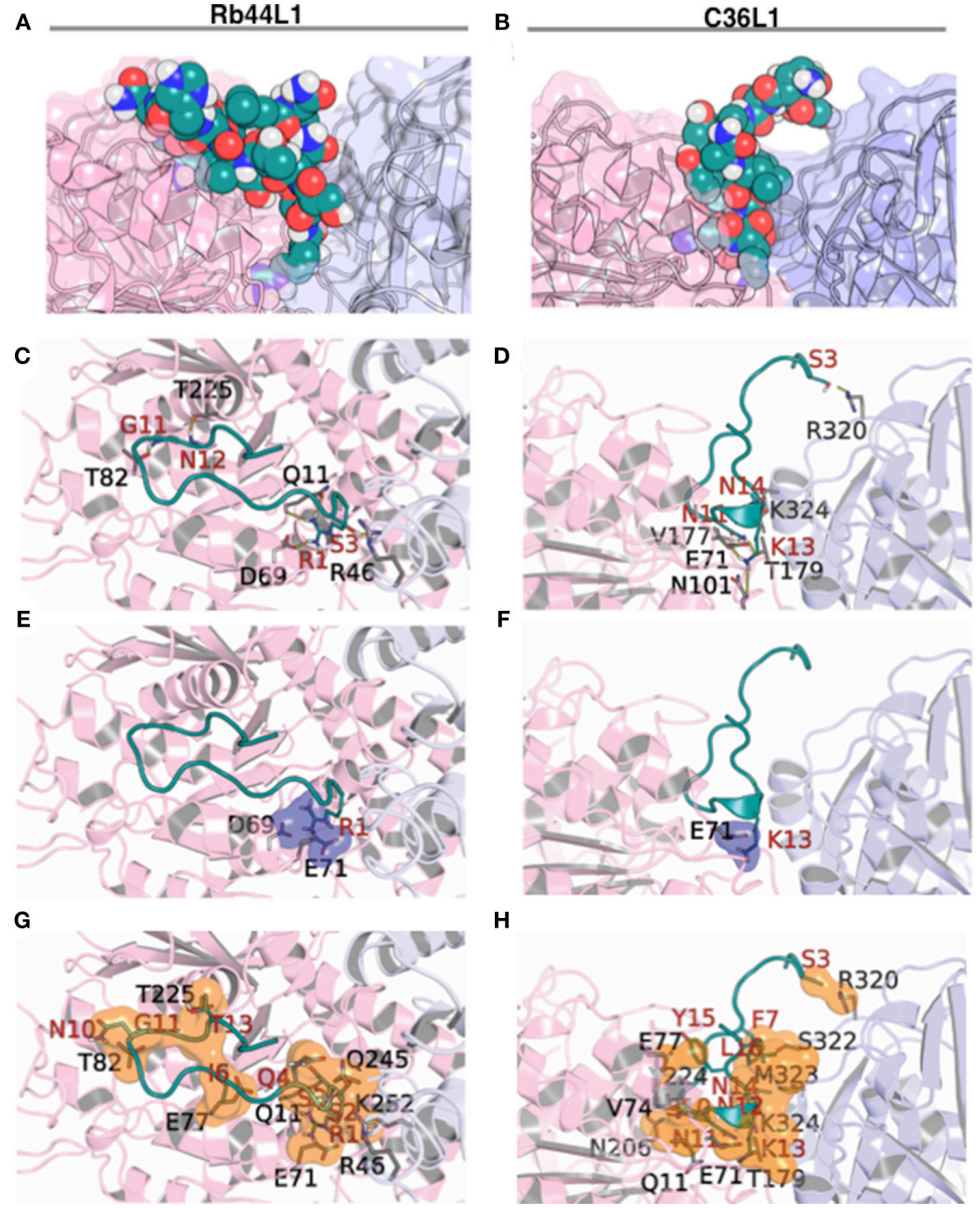
Rb44L1 and C36L1 interactions with α/β-tubulin displaced by 2 Å and 4 Å, respectively. **(A,B)** surface complementarity; **(C,D)** hydrogen bonds formed; **(E,F)** salt bridges form a tiny pocket demonstrated in navy blue surface; **(G,H)** hydrophobic contacts form pockets represented by orange surface. α-Tubulin is represented in light-pink and β-tubulin in light-blue.

Results indicated less favorable interactions for Rb29L1 than C36L1 and Rb44L1 in almost all displacements (as summarized in Table 3, detailed in Table S1, respectively), according to experimental results. At the best pose for Rb44L1 (docked with tubulin displaced by 2 Å) the ^1^R side-chain is buried in the cavity formed between tubulin monomers, participating in 3 of 6 H-bonds and 2 salt-bridges (Figures 7A,C,E,G). In fact, interactions involving ^1^R were observed in all displacements except at 3 Å and 4 Å. This indicates the putative importance of this residue to maintain the interaction with tubulin. When the ^1^R is replaced by alanine, the results showed a systematic worsening of energy values (as summarized in Table 3, detailed in Table S1). Biological assays confirmed this prediction since the R1A substitution in Rb44L1 was not cytotoxic to B16F10-Nex2 cells, in the 0 to 500 μM range (data not shown). C36L1 pose analysis also indicated the involvement of a basic residue governing the interaction with tubulin. The ^13^K was present participating of H-bond, salt-bridge or cation-pi interactions in all tubulin displacements but at 5 Å. At the best pose–docked with tubulin displaced by 4 Å, ^13^K appeared in two H-bonds and in a salt-bridge (Figures 7B,D,F,H). Moreover, its side-chain was also buried in a cavity between tubulin monomers. On the other hand, although Rb29L1 had a greater number of H-bonds, the lack of charged residues would contribute to predicted energies higher than the other peptides.

**Table 3:** Key binding characteristics governing tubulin and L1-CDR interaction.

	**Displacement (Å)**						
	**0**	**1**	**2**	**3**	**4**	**5**	**6**
***Rb29L1***							
Energy^*^	434.76	751.84	383.90	−60.19	800.12	**−101.62**	−37.13
H-bonds	2	3	5	4	8	**1**	8
Salt-bridges	–	1	–	–	–	–	–
Cation-pi	–	–	1	–	–	–	–
T-stacking	–	–	–	–	–	–	1
Hydrophobic contacts	27	69	33	45	57	**60**	58
***C36L1***							
Energy^*^	−41.64	−186.92	15.15	−59.66	**−287.46**	195.54	−271.06
H-bonds	7	2	2	2	**6**	–	4
Salt-bridges	1	1	1	1	**1**	–	–
Cation-pi	–	–	1	–	–	–	1
T-stacking	–	–	–	–	–	–	–
Hydrophobic contacts	60	67	56	45	**42**	59	87
***Rb44L1***							
Energy^*^	216.31	−169.17	**−300.48**	45.49	−70.64	−30.10	−165.28
H-bonds	4	2	**6**	3	1	3	3
Salt-bridges	1	–	**2**	–	–	–	–
Cation-pi	–	–	–	–	–	–	–
T-stacking	–	–	–	–	–	–	–
Hydrophobic contacts	51	57	**61**	47	47	56	64
***Rb44L1-R1A***							
Energy^*^	253.99	45.25	−52.26	35.38	−37.68	−78.29	**−125.58**
H-bonds	2	3	3	–	2	3	**4**
Salt-bridges	–	–	–	–	–	–	–
Cation-pi	–	–	–	–	–	–	–
T-stacking	–	–	–	–	–	–	–
Hydrophobic contacts	41	53	51	46	47	56	**64**

*Best poses are highlighted in bold (energy values in kJ/mol)*.

* *Predicted summed electrostatic energy by atom-type pair according to Gasteiger partial charges*.

### Docking Studies Showed the L1-CDR Interactions Preferentially at the Nonexchangeable Nucleotide-Binding Site

**Figure 8:**
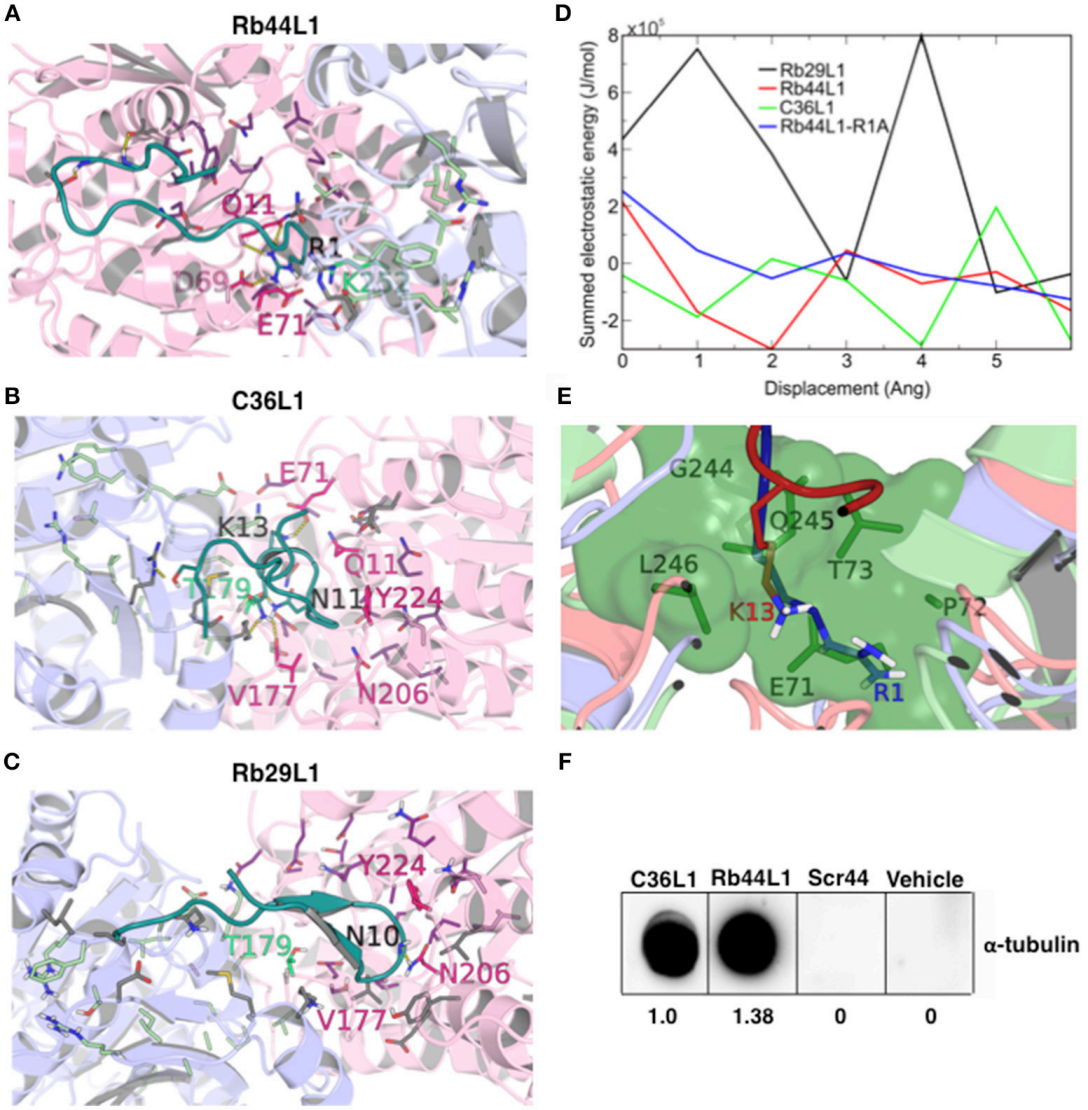
CDR-L1 docking poses in relation to α/β-tubulin N-site and colchicine binding sites and energy re-score of docked complexes. **(A)** Rb44L1; **(B)** C36L1; **(C)** Rb29L1 best docking pose highlighting their position in relation to residues at N-site and colchicine binding sites. α-Tubulin is represented in light-pink and β-tubulin in light-blue. N-site and colchicine binding site residues are represented as purple and pale green, respectively, whereas those that interact with the CDRs are hot pink and lime green for nucleotide and colchicine binding site, respectively. The camera was inverted 170° on the y-axis and 80° on the x-axis for better visualization of the C36L1 and Rb29L1 interactions; **(D)** summed electrostatic energy of Rb29L1, Rb44L1, C36L1 and Rb44L1-R1A complexed with α/β-tubulin at different displacements; **(E)** overlapping of Rb44L1 and C36L1 docked complexes. Residues ^1^R and ^13^K of Rb44L1 (blue) and C36L1 (red), respectively, occupy the same region at the tubulin dimer interface, that is blocked by residues ^70^LEPT of α-tubulin and ^243^PGQL of β-tubulin at minimized structure (green surface); **(F)** Rb44L1 binds to tubulin present in the lysate of B16F10-Nex2 cells. Dot-blottings were performed by coating the nitrocellulose membranes with 10 μg of C36L1, Rb44L1, scrambled-Rb44L1 (Scr44), and vehicle (1% DMSO in milli-Q water). Experimental and control dot-blottings were performed as described in methods. Quantitation of dots was performed using ImageJ, and are represented as arbitrary units.

We evaluated the best docking pose for both Rb44L1 and C36L1 in relation to the exposed nucleotide and colchicine binding sites. Rb44L1 interacted with three residues of the N-site (^11^Q, ^69^D, and ^71^E) and with one residue of the colchicine site (^252^K). The ^1^R participated in all interactions. C36L1, however, interacted with different residues of the N- site (^71^E, ^11^Q, ^224^Y, ^206^N, ^177^V) and one residue of colchicine site (^179^T). These interactions depended on ^13^K and ^11^N residues of the C36L1 peptide (Figures 8A,B). Rb29L1 showed interactions with tubulin similarly with those of C36L1 (^177^V, ^179^T, ^206^N, and ^224^Y). In contrast, there were interactions shared with Rb44L1 and C36L1 (^11^Q and ^71^E), which were absent in Rb29L1 (Figure 8C). Taken together, these results showed that tubulin-opening motion corresponded to a decrease of summed electrostatic energy values of the displaced structures (Figure 8D).

Both Rb44L1 and C36L1 interacted with the region of helices α2, α3 and α8 of α-tubulin subunit, and showed differences in relation to β-tubulin monomer. While Rb44L1 interacts with loops α1β1 and α7α8, C36L1 interacts with loop β9α11 and helix α11. The overlapping of C36L1 and Rb44L1 best poses showed residues ^13^K and ^1^R occupying the same region at the tubulin dimer interface, that is blocked by residues ^70^LEPT of α-tubulin and ^243^PGQL of β-tubulin in a minimized structure (Figure 8E). Rb44L1 interaction with α-tubulin subunit was further confirmed using a chemiluminescence dot-blotting assay. We observed that Rb44L1 significantly bound to α-tubulin present in B16F10-Nex2 cell extract, as compared to the negative control and the scrambled peptide (Scr44), which was inactive. The C36L1 peptide was used as a positive control (Figure 8F). Different concentrations of the coated peptide Rb44L1 were tested and we found 10 μg/10 μl to give the best resolution in the dot-blotting (Figure S4). Interaction with β-actin was also evaluated and no reaction was seen (data not shown). As the docking studies revealed that the Rb44L1 interacted preferentially close to the N-site, we investigated the influence of additional GTP and Mn^2+^ on the peptide binding to α-tubulin in a dot-blotting assay with fixed peptide and melanoma cell lysate as a source of α-tubulin (monomeric, modified, dimeric). The peptide binding was enhanced in the presence of both GTP and Mn^2+^, but not with these agents added separately (Figure S5). Since the GTP N-site is nonexchangeable and non-catalytic, most likely the addition of GTP and Mn^2+^ triggered tubulin assembly by interacting on the E-site. Oligomeric tubulin bound to the peptide explains the increased reactivity with anti-α-tubulin antibody used to reveal the dot-blotting assay.

### Rb44L1 Inhibits Purified Tubulin Assembly

**Figure 9:**
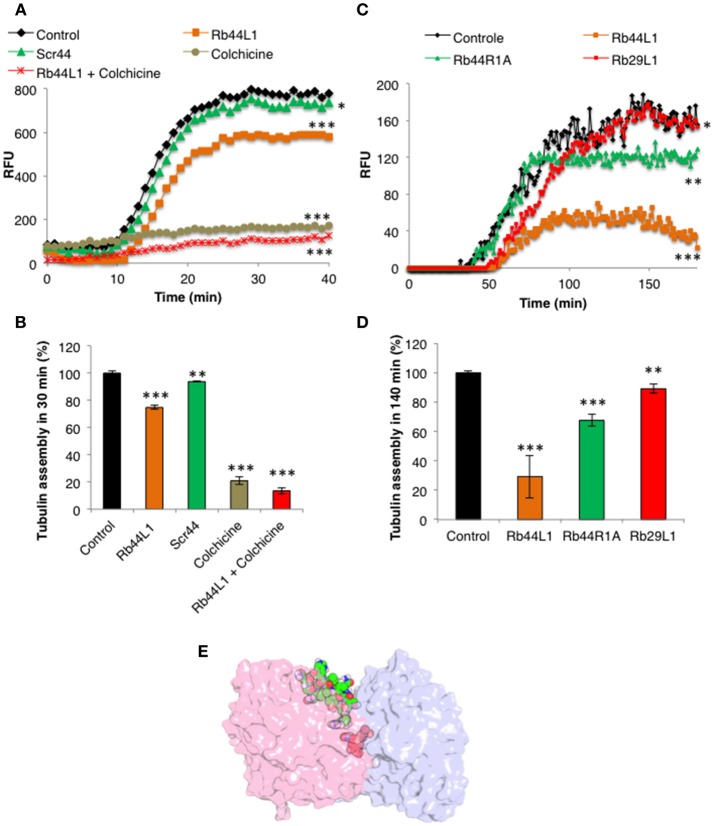
Effects of Rb44L1 on microtubule assembly. **(A)** Polymerization kinetics in presence of Rb44L1 and Scr44 (130 μM) with purified fluorescent tubulin at 2 mg/ml. Inhibition by colchicine (50 μM) was assayed alone or in combination with Rb44L1. **(B)** bar graph represents the percentage of tubulin assembly measured at 30 min from kinetic curve demonstrated in **(A)**. **(C)** Rb44L1, Rb29L1, and Rb44R1A were incubated with tubulin at 1 mg/ml and polymerization/destabilization was measured. **(D)** bar graph represents the percentage of tubulin assembly measured at 140 min from kinetic curve demonstrated in **(C)**. *p < 0.05, **p < 0.01, and ***p < 0.001 in comparison to the control **(E)** structural alignment between the best pose from molecular docking and PDB 4O2B (chains A and B) illustrating the possibility of Rb44L1 (green) and colchicine (red) interact concomitantly with α/β-tubulin at its interface. PDB4O2B was firstly aligned with PDB4TV9, and then the comparison was made. α-Tubulin is represented in light-pink and β-tubulin in light-blue.

The microtubule destabilization effect of Rb44L1 was also evaluated using a fluorescence recombinant tubulin polymerization assay kit (Cystoskeleton, Inc., Denver, CO). In this setting and starting with 2 mg/ml tubulin, 0.2 mg/ml of Rb44L1 delayed tubulin assembly and reduced approximately 1/4 of the total assembly capacity compared to the control (^***^p < 0.001) and the scrambled peptide, Scr44 (Figures 9A,B). This effect was significantly more evident at half the tubulin concentration (1 mg/ml) and 0.2 mg/ml of Rb44L1 (^***^p < 0.001 compared to the control). Polymerization was inhibited in 3/4 followed by depolymerization, after approximately 150 min incubation (Figures 9C,D). Since colchicine is a well-known microtubule inhibitor and has a binding-site mostly on β-tubulin, we assayed the effect of simultaneous addition of colchicine and Rb44L1. Increased inhibition of tubulin assembly was observed with this combination, suggesting independent interaction sites of Rb44L1 and colchicine, ^***^p < 0.001 compared to the colchicine alone (Figure 9E). It should be pointed out that single drugs such as the MT- depolymerizing colchicine and the MT-polymerizing paclitaxel when used in combination, the depolymerization effect has predominated (47).

## Discussion

The microtubules together with various stabilizing and destabilizing molecules display many important physiological functions. Due to their indispensability in the mitotic cell division, microtubules have been selected as preferred anticancer targets. Indeed, microtubule directed drugs are among the most commonly prescribed agents in cancer chemotherapy (2). Recently, anti-tumor peptides targeting microtubules (26) have been studied as tubulin interacting ligands that may evolve to be used in cancer therapy.

Novel anti-tumor peptides may have advantages over mAbs and tyrosine-kinase inhibitors, such as low cost, high specificity and potency due to their compatibility with targeted proteins, ability to penetrate the cell membrane, reduced immunogenicity, and improved safety (48). For example, the ADH-1 (Exherin), is an anticancer peptide distributed by Adhex Technologies®, which targets N-cadherin and induced partial and complete protective responses in patients with metastatic melanoma (49).

The microtubule destabilizing Ig V_L_ CDR1 peptide (C36L1) triggered cytotoxic and cytostatic effects on melanoma cells *in vitro* (23). Besides C36L1, we found that another CDR-L1 derived peptide, from anti-Lewis B mAb, exhibited similar cytotoxic mechanisms, targeting microtubules (MT). In the present work, we studied the molecular structure and biological effects of different L1-CDR-derived peptides: C36L1, Rb44L1 and Rb29L1 on microtubules. We analyzed the structure of L1-CDR-destabilizing MT peptides C36L1 and Rb44L1, as compared to the inactive one, Rb29L1. The latter demonstrated the most stable and rigid structure, assuming a β-hairpin conformation with several high occupancy H-bonds. Rb44L1 showed less rigidity as compared to Rb29L1, with a stable β-bridge conformation, while C36L1 was the most flexible peptide among them.

The biological effects of the peptides were examined and Rb44L1 showed the highest cytotoxic activity, selectively in different cancer cell lines with no significant effects on non-tumorigenic cell lines (Table 2).

Morphological and biochemical changes during tumor cells incubation with cytotoxic concentrations of Rb44L1 were observed. Apoptosis was recognized by the remarkable shrinkage of the cytoplasm, roundup cells with pseudopodia retraction and shriveling without cell lysis, genomic DNA condensation and fragmentation, and exposure of phosphatidylserine at the surface of peptide-treated cells (50). The intrinsic pathway involves the functional deregulation of mitochondria, which may culminate in activation of caspases and the cascade of events that drives to cell death (51, 52). Early disruption of mitochondrial membrane potential, as evidenced by time-lapse fluorescence microscopy and TEM, together with later production of ROS, cleavage of caspase-9, caspase-3, the PARP, upregulation of Bax and downregulation of Bcl-2 were effects induced by Rb44L1, and they are all consistent with the intrinsic pathway of apoptosis (53, 54), strongly suggesting that this is the main *in vitro* cytotoxic mechanism of the peptide in melanoma cells.

P53 is activated in response to different stresses leading tumor cells to apoptosis and growth arrest (55). In this regard, accumulation of active p53 may also be attributed to disintegration of the cytoskeleton. Microtubule targeted-drugs are one of the main stimuli able to increase levels and activate p53 (56).

The main mechanism that seems to be involved in the intrinsic apoptosis by Rb44L1 peptide is the early disruption of the microtubules in melanoma cells. Rb44L1 destabilized labeled microtubules during early stages of incubation, as observed by fluorescence microscopy. In contrast, Rb29L1 did not affect the microtubule dynamics, under the same conditions.

The actin cytoskeleton integrity was also evaluated, as observed by fluorescence microscopy. Rb44L1 induced the degradation of actin filaments in melanoma cells to a maximum effect after 3 h of tumor cell treatment with this peptide. Alterations of actin dynamics are sufficient to induce apoptosis. They involve changes in F-actin levels, in the flux of actin through the filament pool, or both (57). In addition, F-actin depolymerization has been implicated in reduced MMP and elevated ROS production, together with shortening of cell lifespan (58), as observed in melanoma cells treated with Rb44L1. The peptide, however, did not directly interact with F-actin to induce depolymerization as suggested by a late kinetics, which follows microtubule depolymerization. In fact, the actin cytoskeleton integrity has been shown to be highly dependent on the microtubule dynamics (59, 60), which is crucial in tumor cells constantly entering the mitotic program as compared to non-tumorigenic cells (2). Cellular functions depend on the crosstalk between microtubules and actin filaments, in which specific proteins bind to microtubules and actin filaments simultaneously, promoting co-organization and coupled growth of both networks (61). Both cytoskeleton constituents are intrinsically related and rearranged during the progress of apoptosis. Important events are regulated by ROCK kinases that actively regulate the actomyosin contractile ring, a process facilitated by the early disruption of microtubules. Protrusions of the plasma membrane also called apoptotic bodies or blebs, are formed, with subsequent depolymerization of actin filaments (62).

**Figure 10:**
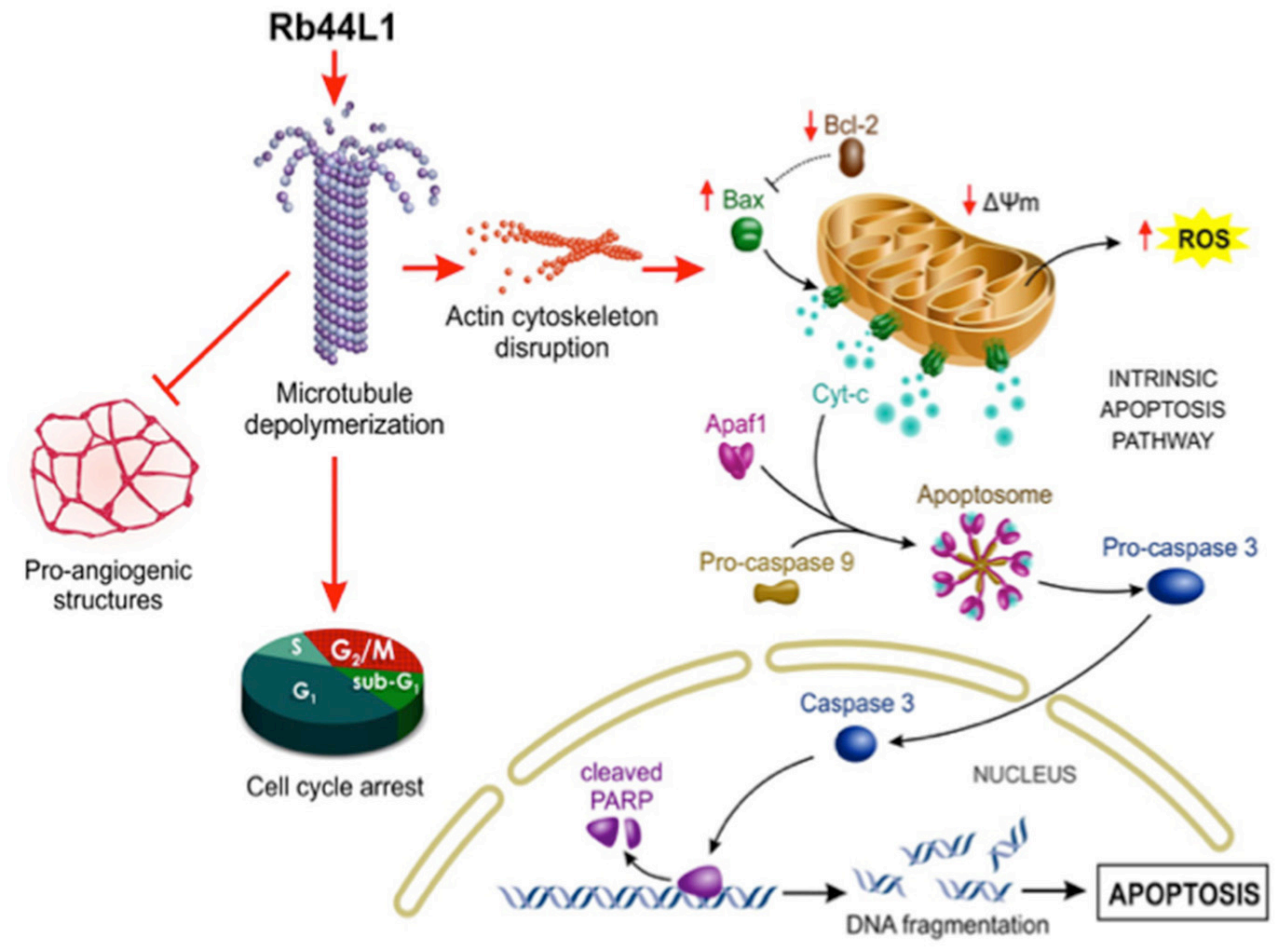
Schematic illustration of proposed Rb44L1 effects on melanoma cells. Rb44L1 peptide interacts at the tubulin monomers' interface in microtubules promoting depolymerization. Alteration of microtubule dynamics led to actin filaments degradation, disrupting the cytoskeleton integrity. In response to changes in the environment, mitochondria produce high amounts of ROS and release co-factors that trigger intrinsic apoptosis. Upon activation by binding to and neutralization of Bcl-2, insertion of Bax into mitochondrial outer membrane form pores to allow the passage of proteins from the intermembrane space to the cytosol. It involves the disruption of mitochondrial membrane potential (Δψm) followed by release of cytochrome c in the cytosol that binds to Apaf-1, ATP, and pro-caspase 9 to form an oligomeric apoptosome, which results in the caspase cascade initiation. Activation of caspase 3 by caspase 9 is responsible for the proteolytic cleavage of the nuclear enzyme Parp-1, which abolishes its DNA repair ability and induces DNA fragmentation in cells undergoing apoptosis. In addition, Rb44L1 inhibited pro-angiogenic structure formation *in vitro* and induced cell cycle arrest at G2/M. Abbreviations: Apaf-1, Apoptotic protease activating factor 1; Cyt-c, Cytochrome-c; ROS, Reactive oxygen species; Δψm, Mitochondrial membrane potential; Bcl-2, B-cell lymphoma 2; Bax, Bcl-2 associated X protein; Parp-1, Poly [ADP-ribose] polymerase 1. The illustration was designed by Carolina de Amat.

Rb44L1 interaction with microtubules and induction of their depolymerization with subsequent degradation of actin filaments increased the number of tumor cells in the G2/M phase leading to a mitotic catastrophe. Such effects, coupled to inhibited angiogenesis as observed *in vitro*, are consistent with the described effects of other microtubule targeting drugs (2, 63). A schematic illustration of the effects induced by Rb44L1 on melanoma cells is detailed in the Figure 10.

Most importantly, this peptide showed a promising antitumor protective effect against subcutaneously grafted melanoma, with no systemic toxicity being observed.

Once proteins exist in equilibrium of multiple conformations in solution, we used a theoretical approach that mixed analyses of molecular dynamics and normal modes, to sample distinct structural states of α/β-tubulin dimer. This hybrid methodology allowed for the assignment of both local and collective motions of the system, that are essential dynamic features related to conformational selection and induced fit, respectively (64–66).

Microtubules are dynamic cellular structures that switch between growing and pruning cycles both *in vivo* and *in vitro*. Stabilization or destabilization of microtubule dynamics is promoted by a number of endogenous and exogenous compounds that regulate the process in different ways, either by competition with GTP (67), structural modification of the protein-protein interface between α and β monomers (8, 31, 68) or by allosteric mechanisms (69). One of the most frequently described mechanisms is the ligand binding at the colchicine site on β-tubulin, which is spatially next to an α-tubulin nucleotide binding site, with nonexchangeable, noncatalytic characteristics, known as N-site. Therefore, we explored the exposition of both binding sites as a molecular docking strategy, since their coupling might trigger the structural destabilization of tubulin dimer exerted by some L1-CDR peptides.

The tubulin heterodimer has two guanine binding sites: at the exchangeable, catalytic site (E-site) on the β chain, GTP is hydrolyzed to GDP during microtubule assembly; the nonexchangeable, noncatalytic site (N-site), on the α chain, is always occupied by GTP, suggesting that it may function as a structural cofactor of tubulin (70). Divalent cations have high affinity for both sites and their binding is associated to the structural stability of tubulin dimer (71). Mg^2+^ is a well-established ion required for microtubule assembly and stability, and contributes to strong GTP binding to the E-site (72). Q-band EPR and electron spin echo envelope modulation spectroscopy showed that Mn^2+^ at both N and E-sites directly coordinated to the triphosphate of GTP, proving that the divalent cation at both sites directly interacts with GTP (73). Mn^2+^ slowly exchanged for Mg^2+^ at the N-site and other divalent and trivalent cations may also exchange at this site and play a role in the assembly of microtubules (74, 75). Chelation of divalent cations in general, inhibits the assembly of tubulin dimers.

L1-CDR peptides bound at the nucleotide/colchicine binding site at the dimer interface, but most of the interactions were made at the N-site. The best solution of Rb44L1 peptide was in an α/β-tubulin semi-open state. We observed that the ^1^R is a key residue for interaction with tubulin dimer. The mutation of this residue for alanine, weakened the interaction, increasing the free energy. This result was further corroborated by experimental assays. Interestingly, the ^13^K of C36L1 used the same tubulin cavity as that of ^1^R of Rb44L1, although C36L1 best docking pose was observed in an open conformation. This polar pocket may play an important role in tubulin depolymerization induced by L1-CDR peptides since the inactive Rb29L1 did not present a favorable interaction on this region.

The inactivity of Rb29L1 peptide is noteworthy, since its sequence is quite similar to Rb44L1 except between residues 4 and 7, which is TSLL in the former peptide and QTIT in the latter. Interestingly, the most favorable docking poses showed a different interaction pattern with tubulin, since Rb44L1 QTIT residues were less solvent exposed than Rb29L1 TSLL residues, which are 70 Å^2^ more exposed to solvent. This is a direct consequence of the observed R interaction pattern with buried tubulin residues (^69^D and ^11^Q) in the N-site, and could be related to the observed activity differences.

A dot-blotting assay showed that in the presence of both GTP and Mn^2+^, but not with these agents added separately, the Rb44L1 peptide bound with increased affinity to the tubulin α-chains of monomeric, modified or dimeric substrates from a tumor cell lysate. This may have occurred by the GTP-E site induced oligomerization of tubulin dimers present in the cell lysate during incubation, indicating that under the conditions used, the dot-blotting assay with fixed peptide did not impair tubulin assembly on the latter (Figure S6).

In contrast, what is the possible mechanism triggering Rb44L1 depolymerization of tubulin? We found that the surface overlapping of the docked conformation of the peptide and the closed α/β-tubulin revealed that the peptide represents a steric constraint to the protein in this conformation. The effect noticed in the overlapping regions of ^1^R, ^3^S, and ^12^N residues, and the peptide size of 1675.0 Å^3^, which preclude the α/β-tubulin return to a closed conformation, is a source of structure destabilization (Video S2).

Taken together, we propose that Rb44L1 peptide is a novel candidate to be developed as a drug, acting on the microtubule network of tumor cells. Molecular docking on tubulin monomers in opening motion, and the possible mechanisms of action leading to microtubule depolymerization were explored in comparison with other Ig CDR-L1 derived peptides, all tested against *in vitro* models of melanoma cells.

## Data Availability Statement

Datasets are available on request. The raw data supporting the conclusions of this manuscript will be made available by the authors, without undue reservation, to any qualified researcher.

## Ethics Statement

This study was carried out in accordance with the recommendations of the Ethics Committee of Federal University of São Paulo, Brazil for animal manipulation and experimental procedures. Animals were provided by Centro de desenvolvimento de modelos experimentais para medicina e biologia (CEDEME), of Federal University of São Paulo. The protocol was approved by the Ethics Committee of Federal University of São Paulo, Brazil via document CEP 1234/2011.

## Author Contributions

NG and CF performed the biological experiments, analyzed data, and designed the figures. MM carried out annexin V and propidium iodide labeling analysis and designed Figure 2D. RA carried out the cell cycle analysis and designed Figure 2E. PR-L and RC carried out all the chemical analysis. NG wrote the manuscript and LP and LT designed the research project. All authors reviewed and approved the manuscript.

### Conflict of Interest Statement

The authors declare that the research was conducted in the absence of any commercial or financial relationships that could be construed as a potential conflict of interest.
