# Supplementary material for: Molecular, Biological and Structural Features of VL CDR-1 Rb44 Peptide, Which Targets the Microtubule Network in Melanoma Cells
Source: Front Oncol. 2019 Jan 25;9:25. doi: 10.3389/fonc.2019.00025 (PMC6355703; doi:10.3389/fonc.2019.00025)
Supplement: Supplementary file 3 [file Data_Sheet_1.docx]

Supplementary Material

**Molecular, biological and structural features of V_L_ CDR-1 Rb44 peptide, which targets the microtubule network in melanoma cells.**

**Natalia Girola^1^, Pedro T. Resende-Lara2, Carlos R. Figueiredo1,3, Mariana H. Massaoka4, Ricardo A. Azevedo1, Rodrigo L.O.R. Cunha^5^, Luciano Polonelli^6^ and Luiz R. Travassos1,7***

^1^Department of Microbiology, Immunology and Parasitology, Experimental Oncology Unit (UNONEX), Federal University of São Paulo, São Paulo, SP, Brazil

^2^Computational Biology and Bioinformatics Laboratory, Federal University of ABC, Santo André, SP, Brazil.

^3^Department of Molecular and Clinical Cancer Medicine, University of Liverpool, Liverpool, UK

^4^Cancer Focus, São Paulo, Brazil

^5^Chemical Biology Laboratory, Natural and Human Sciences Center, Federal University of ABC, Santo André, SP, Brazil.

^6^Unit of Biomedical, Biotechnological and Translational Sciences, Department of Medicine and Surgery, Universitá degli Studi di Parma, Parma, Italy.

^7^Recepta Biopharma, São Paulo, SP, Brazil.

*** Correspondence:**Luiz R. Travassos
luiztravassos@gmail.com

## Supplementary Figures

##
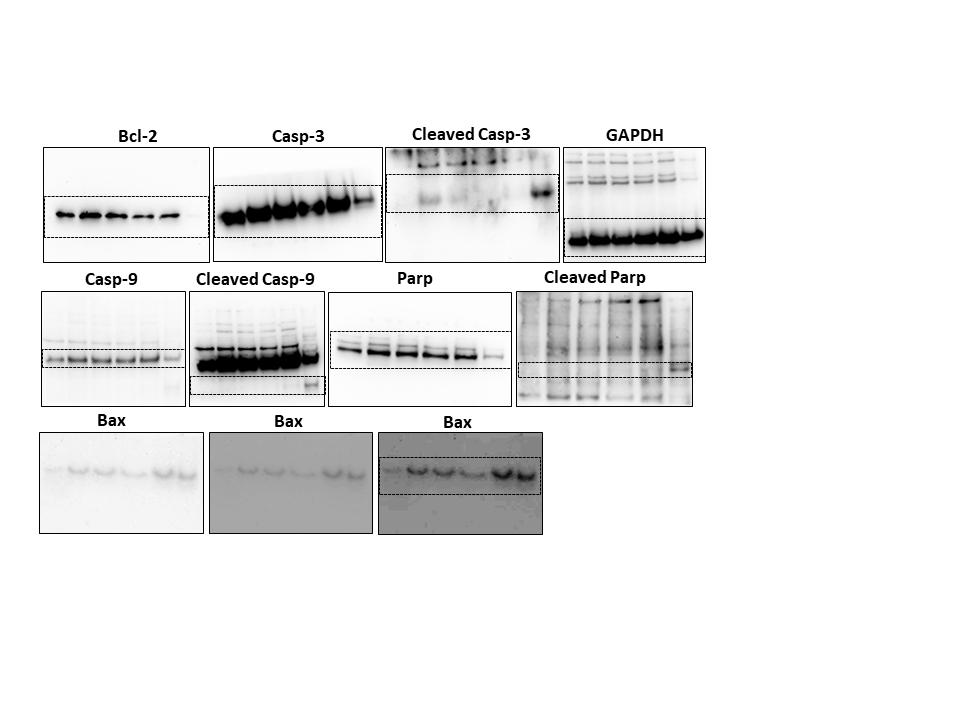


**Supplementary Figure 1:** Uncropped full-length pictures of Western Blotting membranes. Dotted lines delineate the cropped area shown in the main figures. Casp-9 antibody detected total and cleaved fragments. The same membrane was overexposed to reveal the cleaved form. Multiple exposures (increased contrast) of Bax bands are shown.

**
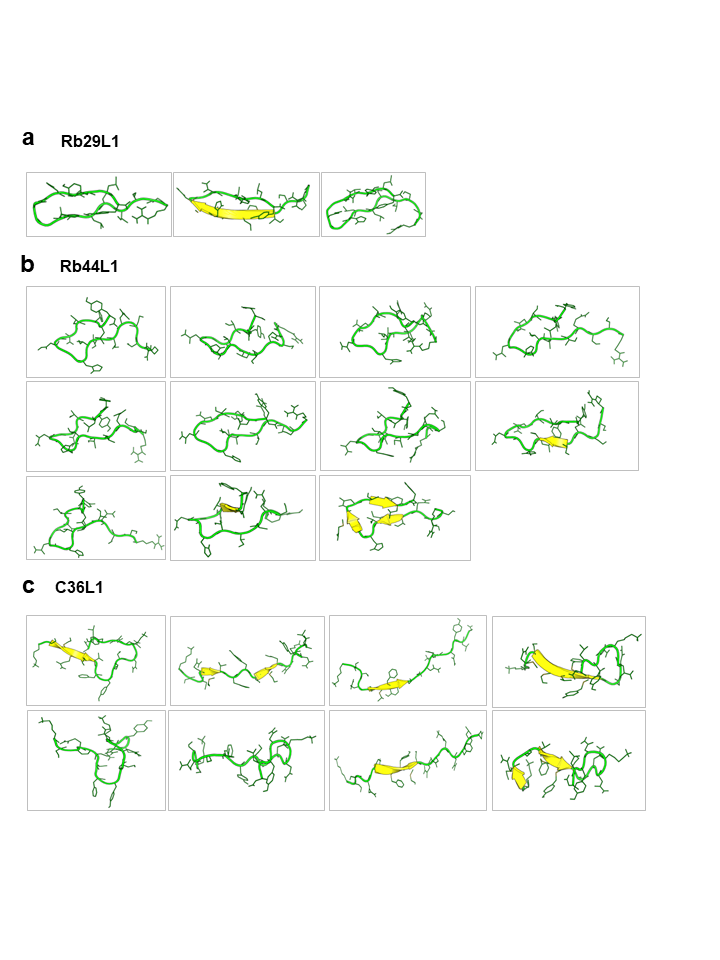
**

**Supplementary Figure 2: Central conformation of each L1 CDR cluster.** (**A**) Rb29L1 clusters; (**B**) Rb44L1 clusters. (**C**) C36L1 clusters.


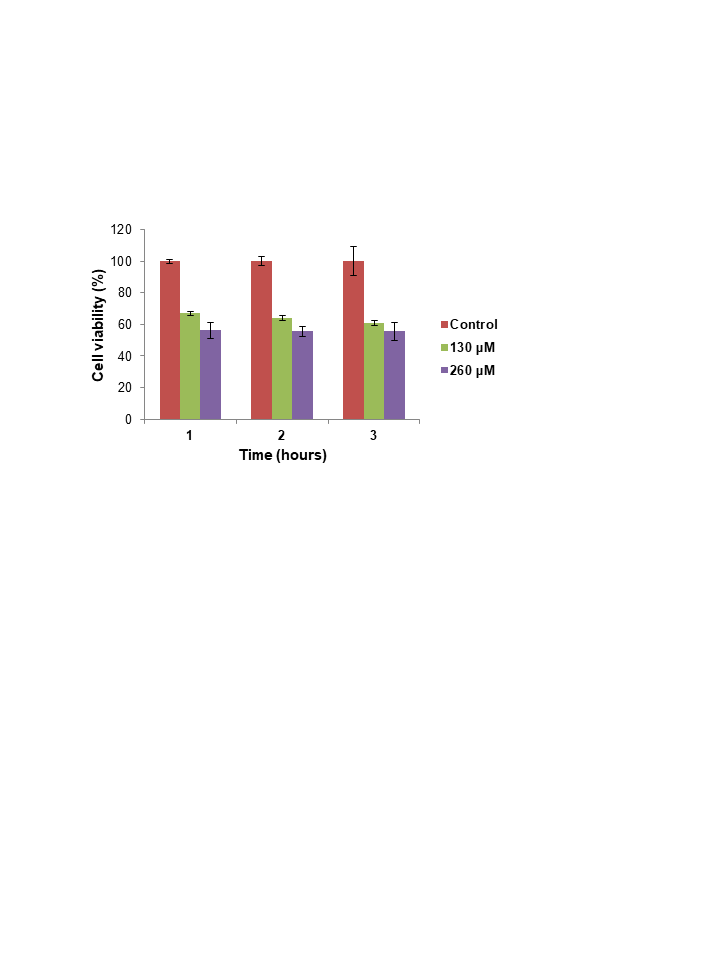


**Supplementary Figure 3: Time-dependent cell viability.** 1x10^4^ B16F10-Nex2 cells were incubated with 130 or 260 µM of Rb44L1 for 1, 2 and 3 h. Viability was measured using MTT colorimetric assay.


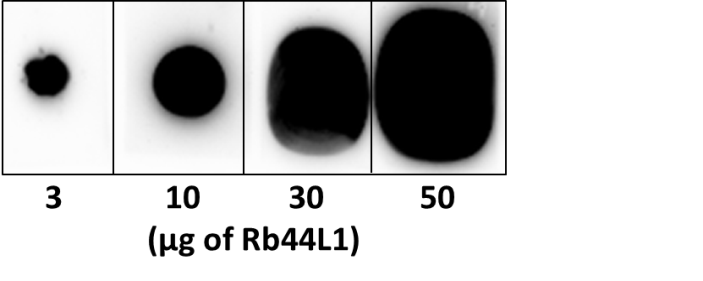


**Supplementary Figure 4:** Dot-blotting analysis testing different concentrations of coated Rb44L1. The membrane was incubated with B16F10-Nex2 cells lysate followed by anti-α-tubulin antibody and anti-rabbit- IgG-HRP.


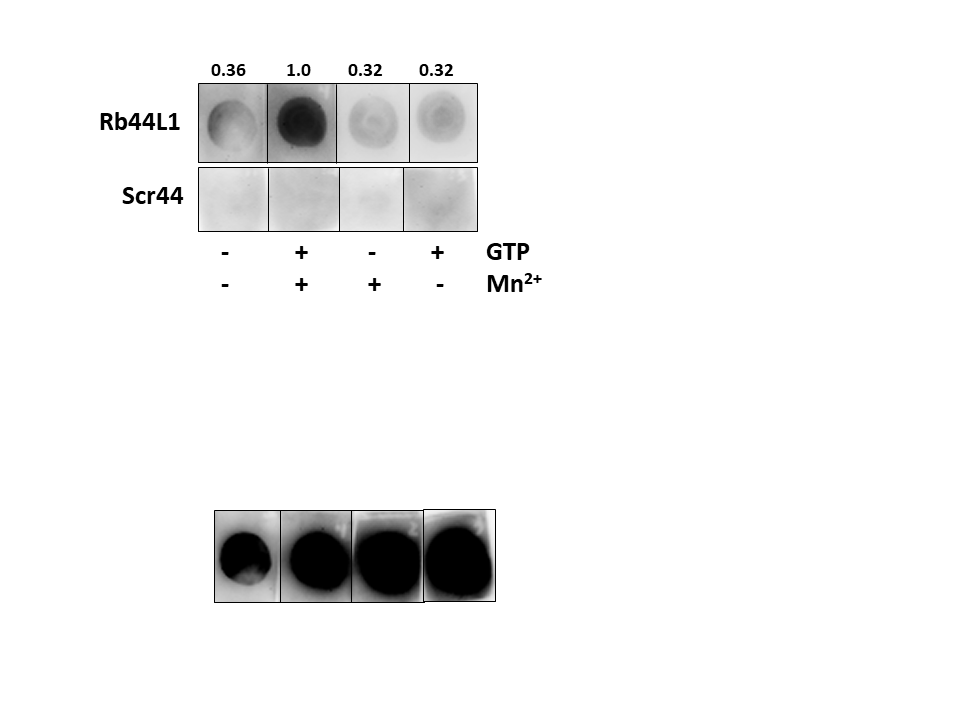


**Supplementary Figure 5:** Binding of Rb44L1 to α-tubulin from total B16F10-Nex2 lysate, with and without 1 mM GTP and/or 1 mM Mn_2_SO_4._H_2_O, both at 10 µg/10 µl on dot-blots of Rb44L1 and scrambled-Rb44L1 (Scr)-coated nitrocellulose membranes. Densitometric analysis of dots was performed using ImageJ. The values are expressed as arbitrary units relative to the signal intensity of Rb44L1 +GTP +Mn^2+^ dot blot.


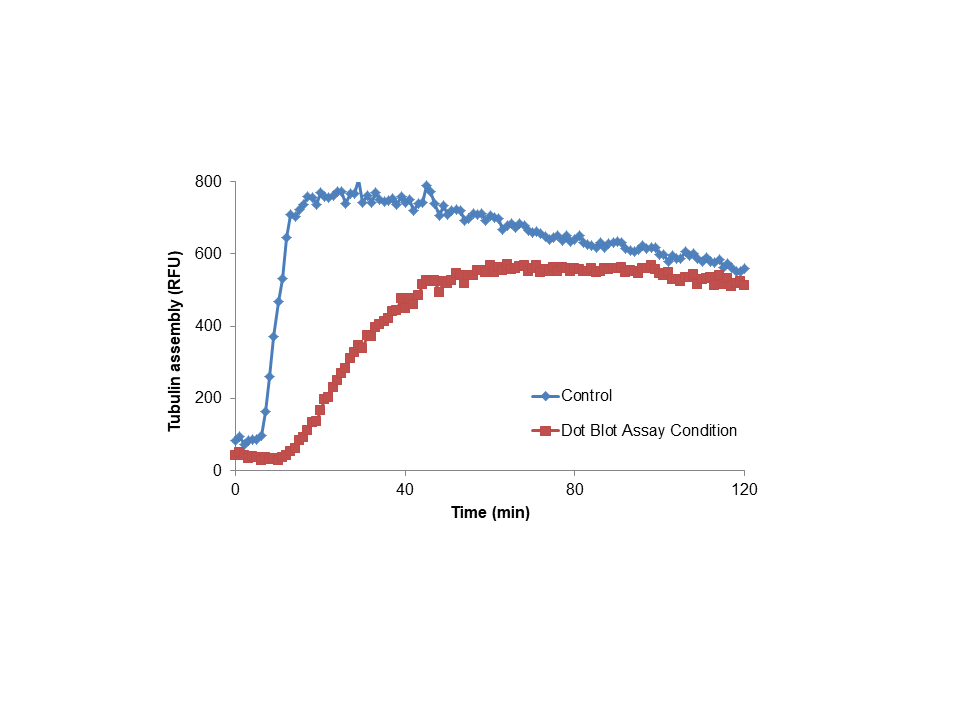


**Supplementary Figure 6:** Fluorescence polymerization kinetics of purified tubulin at 2 mg/ml with or without 0.1% BSA in PBS and 3.4% of cell lysis buffer (dot blot assay condition).

**Supplementary Video legends**

**Supplementary Video 1: Kinetics of peptide induced microtubule depolymerization.** Rb44L1 and Rb29L1 were incubated with CellLight® Tubulin-RFP transduced B16F10-Nex2 cells at 260 µM and compared to the negative control (vehicle). Fluorescent images were taken at 10-min intervals during 2h using a time-lapse BioStation microscope (Nikon, Tokyo) (magnification, ×400). Collected images were used to create the video file with the aid of the NIS-Elements analysis software (Nikon, Tokyo) and Adobe After Effects software.

**Supplementary Video 2: Surface representation of steric clashes between Rb44L1 docked conformer and tubulin in closed conformation**. The best docking solution of Rb44L1 peptide in tubulin was identified in a semi-open state of the latter (with a mass-weighted root-mean square displacement of 2 Å from the crystallographic structure). The surface overlapping between the docked conformation and the closed α/β-tubulin (chains A and B from PDB 4TV9, respectively) reveals that the peptide represents a steric constraint to the protein in the completely closed conformation. This effect can be noticed by the regions were peptide and protein surfaces overlap, notably in the Arg1, Ser3 and Asn12 residues. Rb44L1 is represented in yellow; α- and β-tubulin are shown in green and cyan, respectively.
